# The global landscape and research trend of phase separation in cancer: a bibliometric analysis and visualization

**DOI:** 10.3389/fonc.2023.1170157

**Published:** 2023-06-02

**Authors:** Mengzhu Li, Yizhan Zhang, Jiajun Zhao, Dawei Wang

**Affiliations:** ^1^ Department of Endocrinology, Shandong Provincial Hospital, Shandong University, Jinan, China; ^2^ Department of Endocrinology, Shandong Provincial Hospital Affiliated to Shandong First Medical University, Jinan, China; ^3^ Shandong Key Laboratory of Endocrinology and Lipid Metabolism, Jinan, China; ^4^ Shandong Institute of Endocrine and Metabolic Diseases, Jinan, China; ^5^ Key Laboratory of Endocrine Glucose & Lipids Metabolism and Brain Aging (Shandong First Medical University), Ministry of Education, Jinan, China

**Keywords:** bibliometric analysis, phase separation, cancer, immunotherapy, visualization

## Abstract

**Background:**

Cancer as a deathly disease with high prevalence has impelled researchers to investigate its causative mechanisms in the search for effective therapeutics. Recently, the concept of phase separation has been introduced to biological science and extended to cancer research, which helps reveal various pathogenic processes that have not been identified before. As a process of soluble biomolecules condensed into solid-like and membraneless structures, phase separation is associated with multiple oncogenic processes. However, there are no bibliometric characteristics for these results. To provide future trends and identify new frontiers in this field, a bibliometric analysis was conducted in this study.

**Methods:**

The Web of Science Core Collection (WoSCC) was used to search for literature on phase separation in cancer from 1/1/2009 to 31/12/2022. After screening the literature, statistical analysis and visualization were carried out by the VOSviewer software (version 1.6.18) and Citespace software (Version 6.1.R6).

**Results:**

A total of 264 publications, covering 413 organizations and 32 countries, were published in 137 journals, with an increasing trend in publication and citation numbers per year. The USA and China were the two countries with the largest number of publications, and the University of Chinese Academy of Sciences was the most active institution based on the number of articles and cooperations. *Molecular Cell* was the most frequent publisher with high citations and H-index. The most productive authors were Fox AH, De Oliveira GAP, and Tompa P. Overlay, whilst few authors had a strong collaboration with each other. The combined analysis of concurrent and burst keywords revealed that the future research hotspots of phase separation in cancer were related to tumor microenvironments, immunotherapy, prognosis, p53, and cell death.

**Conclusion:**

Phase separation-related cancer research remained in the hot streak period and exhibited a promising outlook. Although inter-agency collaboration existed, cooperation among research groups was rare, and no author dominated this field at the current stage. Investigating the interfaced effects between phase separation and tumor microenvironments on carcinoma behaviors, and constructing relevant prognoses and therapeutics such as immune infiltration-based prognosis and immunotherapy might be the next research trend in the study of phase separation and cancer.

## 1 Introduction

Cancer, a complex disease characterized by uncontrolled cell proliferation, is one of the leading causes of human death (1, 2). Although much work has been done to decipher the underlying mechanisms behind cancer development, the pathogenesis of this severe disease remains to be investigated because the development and progression of cancer is a dynamic process involving multiple gene-environment interactions (1–3). Recently, the concept of phase separation has been introduced into cancer research, which dramatically expands our knowledge of cancer biology (4–6).

Phase separation is the process of separating (or semi-separating) a homogeneous liquid solution (or phase) of macro-molecular components such as proteins and nucleic acids into two distinct phases, one enriched in macromolecules and the other depleted of the same macromolecules (7). The cellular structures formed by phase separation are called membraneless organelles or biomolecular condensates to reflect their origin through condensation formation (8). Unlike canonical membrane-bound organelles such as the nucleus, mitochondria, and endoplasmic reticulum, these membraneless structures are not enveloped by a lipid bilayer, and thereby they are inherently unstable, allowing them rapidly assemble or disassemble to satisfy cell requirements (8, 9). Biomolecular condensates provide a capacity to concentrate certain molecules in stable and well-defined structures, resulting in high biochemical reaction rates; for instance, the RNA cleavage activity of hammerhead ribozyme could be increased by 70 folds in phase-separated droplets (10–12). To date, biomolecular condensates are found to participate in various cellular processes such as genomic regulation, signal transduction, and protein degradation (8).

Interestingly, reciprocal causation is observed between abnormal phase separation and cancer development. Phase separation aberrations are closely associated with several features of cancer, including sustained proliferative signaling, inducing angiogenesis, and cell death resistance (13, 14). For example, elevated AKAP95 expression leads to membraneless structure formation in cancer, which could promote cancer outgrowth by controlling the adequate splicing of cancer-related targets (15); by binding to super-enhancers, Myc forms transcription condensates to enhance VEGF expression, leading to the promotion of angiogenesis (16); the ability of tumor cells to resist cell death under stress conditions is partially ascribed to their prompt formation of stress granules, which are another kind of membraneless organelles responsible transient translation halting for cell energy saving and life prolonging (17). On the other hand, cancer-associated mutations could initiate the formation of new biomolecular condensates, which would activate oncogenic pathways or drive oncogene transcriptions to promote tumorigenesis (18, 19). For example, SHP2 cancer-related mutants are prone to forming phase-separated compartments that boost oncogenic MAPK hyperactivation (18). Interestingly, small‐molecule compounds such as ET516 and Elvitegravir, which could respectively disrupt the condensate formation of androgen receptors, and SRC-1/YAP/TEAD, have already exhibited anticancer effects (20, 21). Therefore, it is of great potential to develop drugs targeting abnormal phase separation for cancer therapeutics.

Bibliometric analysis, a valuable tool for scientific researchers, could comprehensively analyze basic information such as the authors, countries, institutions, journals, and citations in particular publications (22). The bibliometric analysis could not only present the hotspots and features of global research but also forecast the direction of future studies. Although phase separation is one of the hot topics in cancer research, there is no bibliometric analysis in this area. In this study, we carried out a comprehensive analysis of the characteristics and hotspots regarding phase separation in cancer from a bibliometric viewpoint, which not only provides a general overview and development trend but also identifies new clues and ideas in this field.

## 2 Methods

### 2.1 Database and searching strategy

The Web of Science Core Collection (WoSCC) was used to search for literature on phase separation in cancer. The paper retrieved was satisfied with the following criteria: {(Topic = neoplas* OR Topic = cancer* OR Topic = Tumor* OR Topic = Malignan*) AND (Topic = phase separation OR Topic = biomolecular condensate* OR Topic = membraneless organelle*)}. The publication period was set from 1 January 2009 to 31 December 2022, and only articles published in English were included. ‘*’ represents any group of characters, including no character.

**Figure 1:**
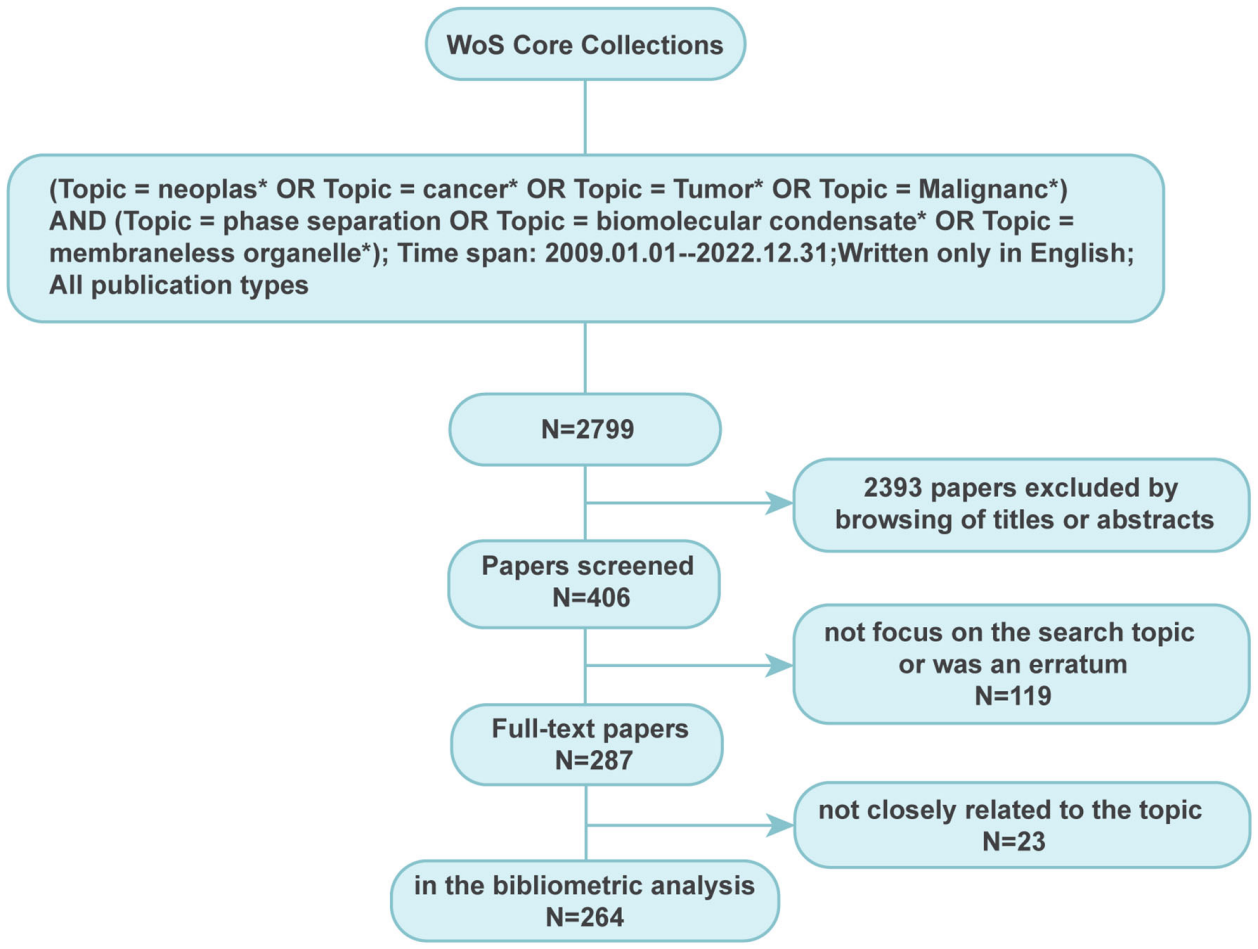
Searching strategy for paper extraction focussing on phase separation in cancer. A total of 264 publications closely related to the topic of phase separation and cancer were finally extracted.

The strategy for paper extraction is illustrated in **Figure 1**. After literature screening, all document types were included except editorial materials such as correspondence, research highlights, and meeting abstracts. All recorded data of selected papers were downloaded from WosCC in the EndNote Desktop format. The paper information such as author names, institutions, countries, and keywords was normalized to a standard format. Duplicate authors were cross-checked among documents to avoid ambiguity.

Ethics committee approval was not applicable in this study as the collected data were all from a public resource.

### 2.2 Data visualization

GraphPad Prism 8 was applied to present the number of papers published at the indicated time. The top 10 authors/organizations/countries and most cited articles were summarized with Microsoft Excel 2021. The bibliometric investigation, including co-authorship analysis, keyword burst, and keyword co-occurrence analysis, was carried out through VOSviewer software (version 1.6.18) and Citespace software (Version 6.1.R6), correspondingly.

## 3 Results

### 3.1 Publication trend and research focus

**Figure 2:**
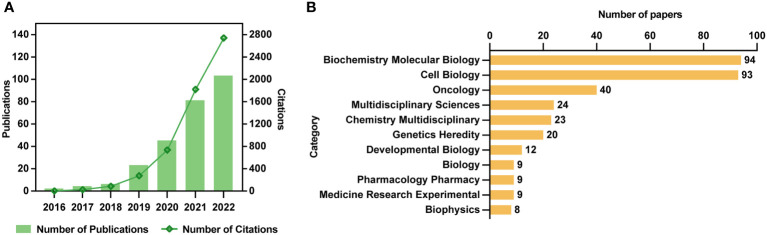
Trends and categories of publications regarding the phase separation in cancer. **(A)** The annual output and citations of papers related to phase separation and cancer from 2016 to 2022. **(B)** Categories of 264 publications.

After literature extraction based on our strategy, 264 papers, comprised of 137 articles and 127 reviews, were enrolled for deep analysis (**Supplementary Material**). It was found that although Clifford P. Brangwynne and colleagues first introduced the concept of phase separation in biology in 2009 (23), the first study on phase separation in cancer was observed in 2016, followed by a yearly rise in the number of publications and citations, as shown in **Figure 2A**. The evolution of the number of publications and citations on phase separation-related cancer research can be divided into two stages. The first stage was from 2016 to 2018, with a slow and steady growth rate. The second phase was from 2018 to 2022, and there had been a spurt in the number of publications and citations, with each year twice as many as the previous year, reaching more than 100 publications and 2800 citations in 2022. Although the discovery of the relationship between phase separation and cancer was less than a decade old, its rapid progress showed that it had a broader scope for future development.

As shown in **Figure 2B**, although these 264 papers were divided into 33 research categories, only seven categories had more than ten publications. Notedly, the categories of biochemistry molecular biology, and cell biology alone accounted for 187 papers (54.8%) in total, indicating the essential physiological functions that phase separation played in cancer cells. The remaining predominant categories, such as Oncology (n=40), Chemistry Multidisciplinary (n=24), Multidisciplinary Sciences (n=23), Genetics Heredity (n=20), and Developmental Biology (n=12), reflected the multidisciplinary cross during the development of phase separation-related cancer research.

### 3.2 Citation counts

There are 6573 citations for these 264 papers, averaging 24.90 per paper. The ten most cited documents related to phase separation and cancer were illustrated in **Table 1**, including four research articles and six reviews. According to the number of citations, the review written by Alberti S. (290 citations) was ranked first, followed by reviews written by Guccione E. (222 citations), Aguzzi A. (215 citations), Stadhouders R. (209 citations), and Lafontaine DLJ. (201 citations). The research articles from the ten most cited papers were respectively written by Bouchard JJ., Nair SJ., Klein IA., and Isoda T., with citations ranging from 184 to 154.

**Table 1:** Top 10 most cited papers related to phase separation and cancer.

Rank	Title	Journal	Type	Authors	Year	Citations
1	Liquid-Liquid Phase Separation in Disease	*Annual Review of Genetics*	Review	Alberti S., et al. (24)	2019	290
2	The regulation, functions and clinical relevance of arginine methylation	*Nature Reviews*	Review	Guccione E., et al. (25)	2019	222
3	Phase Separation: Linking Cellular Compartmentalization to Disease	*Trends in Cell Biology*	Review	Aguzzi A., et al. (4)	2016	215
4	Transcription factors and 3D genome conformation in cell-fate decisions	*Nature*	Review	Stadhouders R., et al. (26)	2019	209
5	The nucleolus as a multiphase liquid condensate	*Nature Reviews*	Review	Lafontaine DLJ., et al. (9)	2020	201
6	Cancer Mutations of the Tumor Suppressor SPOP Disrupt the Formation of Active, Phase-Separated Compartments	*Molecular Cell*	Article	Bouchard JJ., et al. (27)	2018	184
7	Phase separation of ligand-activated enhancers licenses cooperative chromosomal enhancer assembly	*Nature Structural and Molecular Biology*	Article	Nair SJ., et al. (28)	2019	164
8	p62/SQSTM1-steering the cell through health and disease	*Journal of Cell Science*	Review	Sanchez-Martin P., et al. (29)	2018	164
9	Partitioning of cancer therapeutics in nuclear condensates	*Science*	Article	Klein IA., et al. (30)	2020	159
10	Non-coding Transcription Instructs Chromatin Folding and Compartmentalization to Dictate Enhancer-Promoter Communication and T Cell Fate	*Cell*	Article	Isoda T., et al. (31)	2017	154

### 3.3 Analysis of countries and organizations

**Figure 3:**
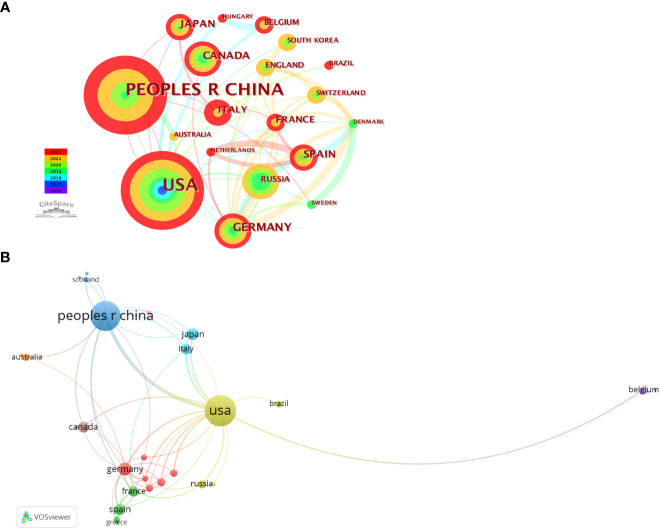
Visual maps of countries related to phase separation and cancer. **(A)** Visualization of country collaborations with Citespace. Node size represents the number of publications. Colors indicate different publish years. **(B)** Country cooperation analysis conducted with VOSviewer. Node size means the count of publications. Line thickness indicates connection strength.

Thirty-two countries had published papers related to this field; the analysis of country distribution revealed that the USA ranked first with 105 articles, accounting for 39.77% of the total publications, sequentially followed by China (n=95, 35.98%), Germany (n=18, 6.82%), Japan (n=15, 5.68%), Spain (n=13, 4.92%), and Canada (n=13, 4.92%) (**Table 2**). The USA and China appeared to dominate this field, with production covering approximately three-quarters of all publications. However, their average number of citations per paper was lower than that of Germany, which ranked first with an average of 60.11 citations per paper. With the criteria of at least three publications from each country, a co-authorship network map was established to evaluate the status of cooperation between countries. It was shown that the USA, the earliest country involved in this field, was the best at cooperating with other countries, followed by Germany (**Figure 3**).

**Table 2:** Top 10 countries ranked by the number of papers related to phase separation in cancer.

Rank	Name	Publications	Percentage	Total citations	Average citations	H-index
1	USA	105	39.77%	3633	34.60	35
2	China	95	35.98%	1217	12.81	18
3	Germany	18	6.82%	1082	60.11	10
4	Japan	15	5.68%	387	25.80	8
5	Spain	13	4.92%	669	51.46	7
6	Canada	13	4.92%	549	42.23	8
7	France	11	4.17%	187	17.00	7
8	Italy	11	4.17%	166	15.09	5
9	Belgium	8	3.03%	266	33.25	6
10	England	8	3.03%	200	25.00	7

**Figure 4:**
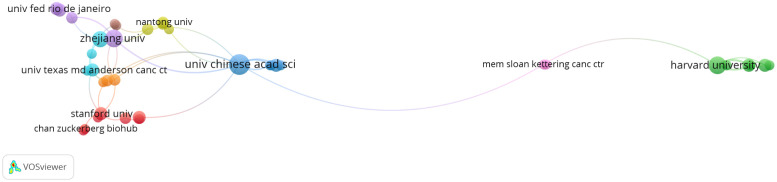
Analysis of organization cooperation. The picture was conducted with VOSviewer. Node size indicates the number of publications. The lines represent cooperation between different organizations.

These 264 papers were generated from 413 different institutions, and the number of papers from the top ten organizations accounted for 38.95% (104/264) (**Table 3**). The most productive organization was the University of Chinese Academy of Sciences (n=16, with 363 citations), followed by the University of Texas System (n=15, with 329 citations). Among these top 10 organizations, according to the average citation (AC), Howard Hughes Medical Institute and Barcelona Institute of Science and Technology ranked the first (AC=80.44) and second (AC=76.5), respectively. Network analysis was performed to analyze the inter-institutional cooperation after the enrolment of organizations with a minimum of three documents, and it was illustrated that the University of Chinese Academy of Sciences was the most active in cooperating with other institutions, followed by Zhejiang University (**Figure 4**).

**Table 3:** Top 10 organizations ranked by the number of papers related to phase separation in cancer.

Rank	Organization	Countries	Publications	Citations	Average citations (AC)	Rank by AC	H-index
1	University of Chinese Academy of Sciences	China	16	363	22.69	7	7
2	University of Texas System	USA	15	329	21.93	6	8
3	University of California System	USA	14	757	54.07	3	10
4	Howard Hughes Medical Institute	USA	9	724	80.44	1	8
5	Harvard University	USA	9	473	52.56	4	8
6	Zhejiang University	China	9	103	11.44	9	5
7	Barcelona Institute of Science and Technology	Spain	8	612	76.50	2	5
8	St Jude Children’s Research Hospital	USA	8	386	48.25	5	7
9	UDICE-French Research Universities	France	8	163	20.38	8	4
10	Sun Yat Sen University	China	8	69	8.63	10	5

### 3.4 Analysis of journals and authors

A total of 137 journals published 264 papers on phase separation in cancer, and the top ten journals with five or more publications were listed in **Table 4**. These journals included 71 articles, accounting for 26.89% of all accepted papers. In contrast, 87 journals (63.5%) only published one paper in the past. The most productive journal is *Molecular Cell*, which published ten articles with the highest H-index (n=9). *Cell* Journal had the highest total citations with only five publications, consistent with its high journal impact.

**Table 4:** List of journals with five or more papers focused on phase separation and cancer.

Rank	Journals	Counts	Percentage	Times Cited	Country	H-index	IF (2021)
1	*Molecular Cell*	10	3.79%	387	USA	9	19.328
2	*International Journal of Molecular Sciences*	9	3.41%	82	USA	5	6.208
3	*Nucleic Acids Research*	8	3.03%	115	England	5	19.16
4	*Frontiers in Cell And Developmental Biology*	8	3.03%	81	Switzerland	5	6.081
5	*Nature Communications*	7	2.65%	101	England	5	17.694
6	*Frontiers in Oncology*	7	2.65%	17	Switzerland	3	5.738
7	*Frontiers in Genetics*	6	2.27%	58	Switzerland	4	4.772
8	*Cancers*	6	2.27%	7	Switzerland	1	6.575
9	*Cell*	5	1.89%	397	USA	5	66.85
10	*Journal of Cell Science*	5	1.89%	270	England	4	5.235

**Figure 5:**
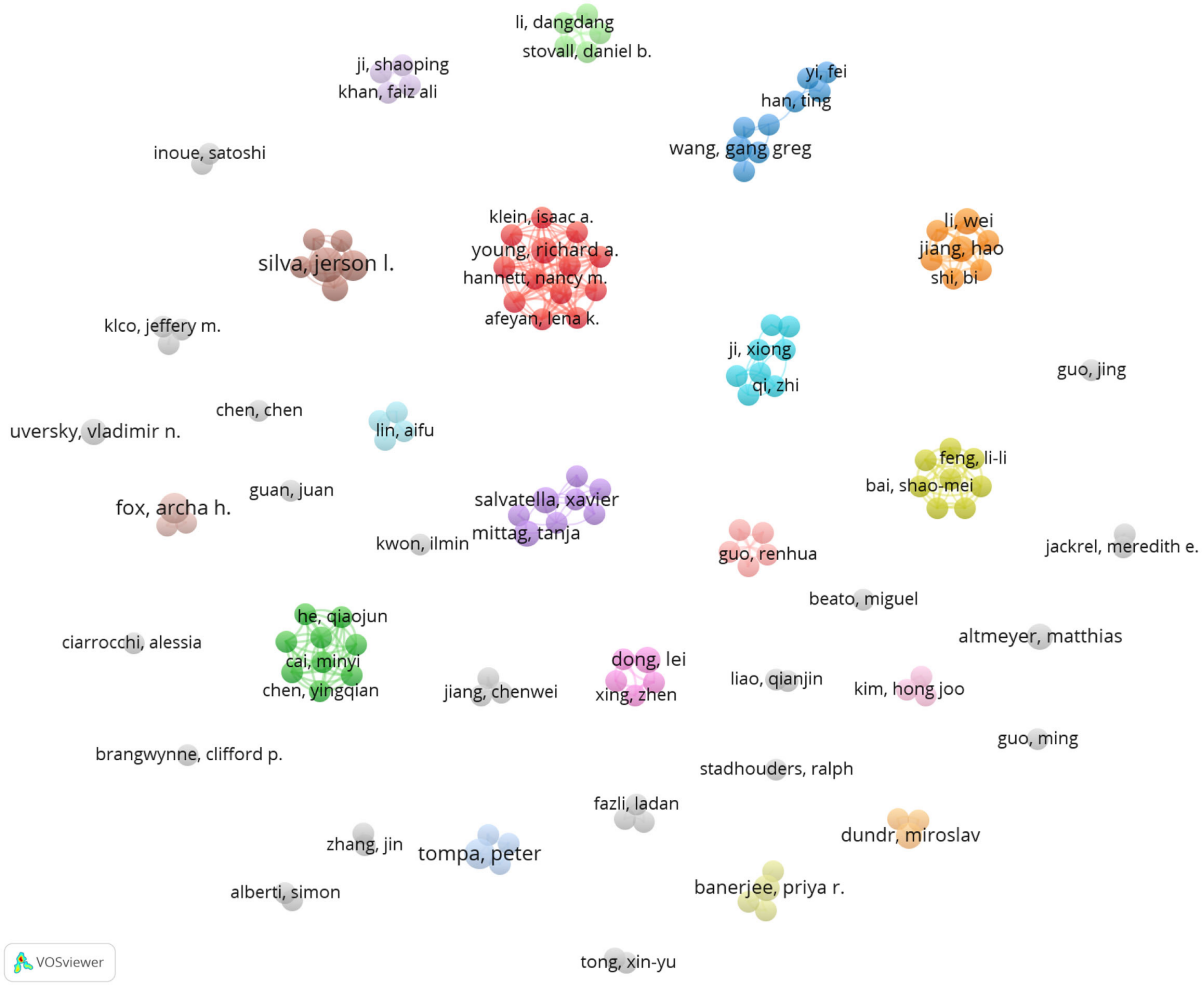
Author analysis in the field of phase separation-related cancer studies. Co-authorship network analysis was conducted with VOSviewer. Different colors mean different clusters. The lines indicate cooperation between the authors.

There were 1677 authors contributing to these 264 publications. Interestingly, it was found that no scholars dominated this area in terms of publication numbers since the most productive author Fox AH, De Oliveira GAP, and Tompa P had published only four articles, respectively (**Table 5**). Surprisingly, Altmeyer M from the University of Zurich achieved the top citations (n=246) with only three papers. An authorship network analysis was then conducted on 119 authors with at least two publications. The 119 authors were then scattered into 36 clusters without obvious connections (**Figure 5**), indicating few author collaborations in this field.

**Table 5:** Top 10 authors ranked by the number of papers related to phase separation and cancer.

Name	Institution	Number of papers	Times of citations	H-index
Fox AH	University of Western Australia	4	116	3
De Oliveira GAP	Universidade Federal do Rio de Janeiro	4	70	3
Tompa P	Flanders Institute for Biotechnology (VIB)	4	32	2
Altmeyer M	University of Zurich	3	246	3
Young RA	Massachusetts Institute of Technology (MIT)	3	244	3
Mittag T	St Jude Children’s Research Hospital	3	228	3
Salvatella X	Barcelona Institute of Science & Technology	3	212	2
Banerjee PR	State University of New York (SUNY) System	3	170	2
Wang GG	University of North Carolina	3	173	3
Dundr M	Rosalind Franklin University Medical & Science	3	124	3

### 3.5 Keyword co-occurrence and burst analysis

**Figure 6:**
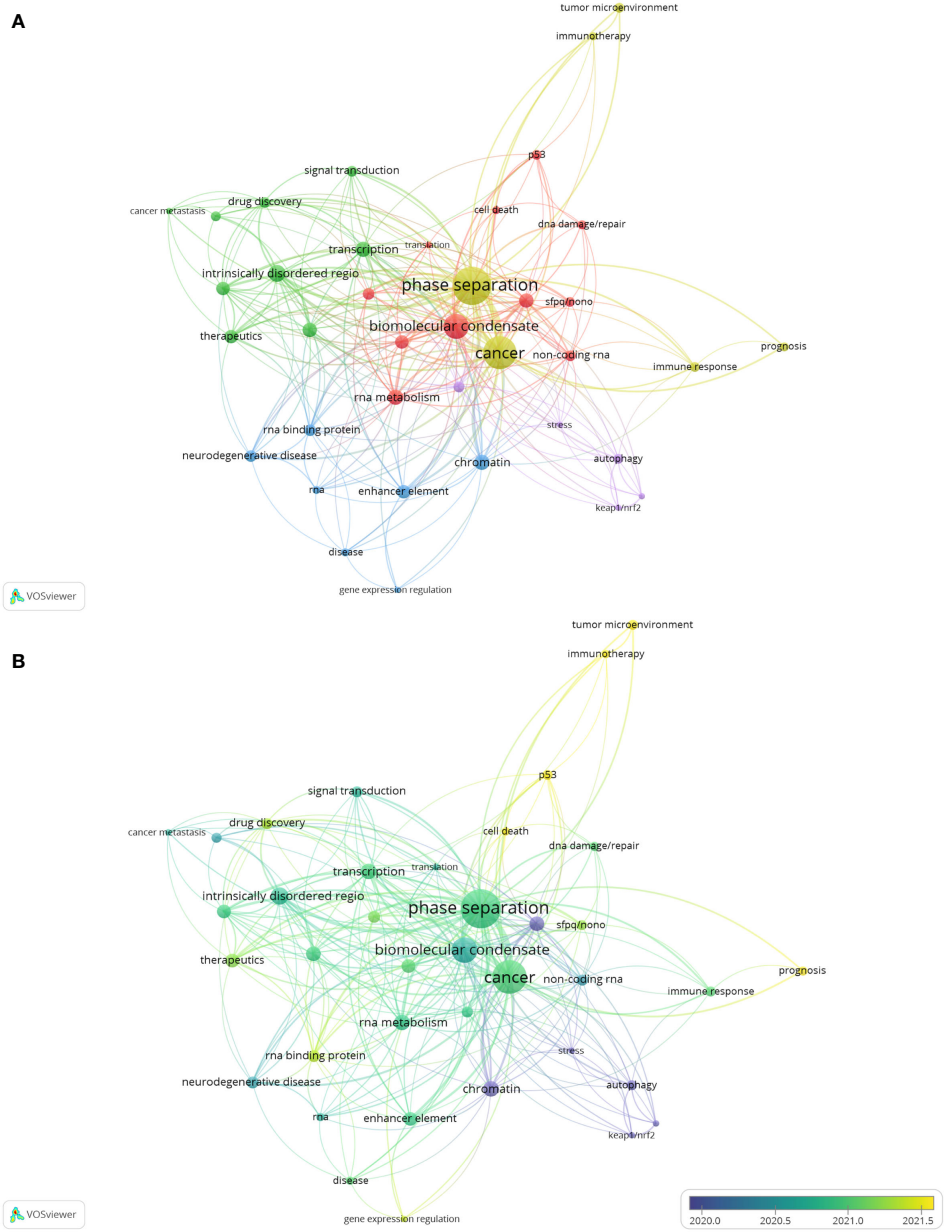
The analysis of keyword co-occurrence. **(A)** A network based on 38 author keywords. Node size indicates the counts of publications. Different colors represent different clusters. **(B)** The overlay visualization map of keywords. The circle size shows the number of publications; The circle colors represent the average published year.

Keyword co-occurrence analysis is a method to evaluate the appearance frequency and associations of two keywords if they are simultaneously present in an article. After visualization *via* VOSviewer, the popularity of keywords and the strength of their interconnections could be indicated by node size and line thickness, respectively. To further investigate the research focus and trends of phase separation in cancer, we applied keyword co-occurrence analysis after merging keywords with the same meanings or categories and collecting 402 keywords in sum from these 264 papers. Thirty-eight keywords satisfied the criterion when an author keyword was limited to 3 co-occurrences. The keyword of “phase separation”, which appeared in 94 papers (n=94), was the most frequent co-occurrence word, followed by “cancer” (n=68), and “biomolecular condensate” (n=39). Then, based on these 38 author keywords, the network was established, which could be further divided into five clusters with different colors (**Figure 6A** and **Table 6**). Cluster 1 (red) contained eleven items: biomolecular condensate, RNA metabolism, nuclear body, RNA granules, epigenetic regulation, non-coding RNA, p53, SFPQ-NONO, DNA damage or repair, cell death, and translation. Cluster 2 (green) contained nine items: intrinsically disordered regions, transcription, post-translational modification, transcription factor, therapeutics, drug discovery, signal transduction, protein-protein interactions, and cancer metastasis. Cluster 3 (blue) contained seven items: chromatin, enhancer elements, neurodegenerative diseases, RNA-binding protein, RNA, disease, and gene expression regulation. Cluster 4 (yellow) had six items: phase separation, cancer, immune response, tumor microenvironment, prognosis, and immunotherapy. Cluster 5 (purple) contained five items: mutations, autophagy, stress, Keap1-Nrf2, p62, and SPOP.

**Table 6:** The clusters organized by keyword co-occurrence analysis.

Clusters	Research hotspots	Number of items	Keywords
Cluster 1	The role of biomolecular condensates in nuclear homeostasis	11	biomolecular condensate, RNA metabolism, nuclear body, RNA granule, epigenetic regulation, non-coding RNA, p53, SFPQ-NONO, DNA damage or repair, cell death, translation
Cluster 2	anticancer drug targeting phase separation process	9	intrinsically disordered regions, transcription, post-translational modification, transcription factor, therapeutics, drug discovery, signal transduction, protein-protein interactions, cancer metastasis
Cluster 3	The role of DNA and RNA elements in phase separation and diseases	7	chromatin, enhancer elements, neurodegenerative diseases, RNA binding protein, RNA, disease, gene expression regulation
Cluster 4	Phase separation in tumor microenvironment-related prognosis and therapy	6	phase separation, cancer, immune response, tumor microenvironment, prognosis, immunotherapy
Cluster 5	How cells cope with internal and external stress by phase separation	5	mutations, autophagy, stress, Keap1-Nrf2, p62, SPOP

To investigate the future directions of phase separation-related cancer research, an overlay visualization map was constructed according to the mean publication year (**Figure 6B**). Purple nodes indicated keywords appearing comparatively earlier, while yellow nodes told those appearing most recently. It was found that earlier research in this field mainly focused on the topics in Cluster 5; however, items in Cluster 4 gradually received more attention with time. Specifically, the keywords of “tumor microenvironment”, “immunotherapy”, “prognosis”, “p53” and “cell death”, with relatively later mean publication years and lower mean frequency of occurrence, were probably the next hotspots in the future.

**Figure 7:**
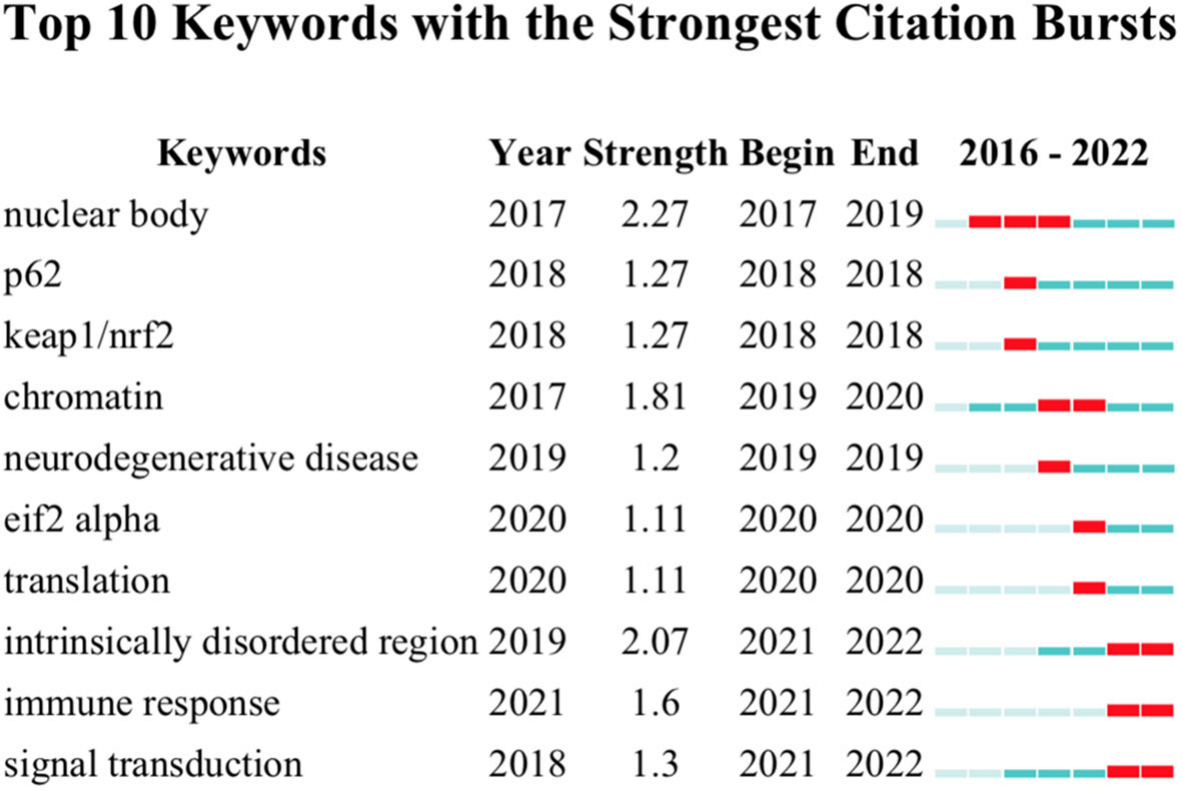
Keyword burst analysis. The graph was conducted with Citespace. Red squares indicate the burst duration.

In addition to keyword overlay visualization, burst keywords, which represent frequently cited words in related research filed over a period of time, could also be used to uncover new research frontiers. After performing a burst keywords analysis *via* Citespace (**Figure 7**), it was found that “nuclear body”, the most extended burst keyword with a duration of 3 years from 2017 to 2019, happened to be the strongest burst with a strength of 2.27, which indicated that elucidating the function and mechanisms of the nuclear body during cancer development was the research hotspot at the initial stage of this research field. Apart from that, keywords including “intrinsically disordered region”, “immune response”, and “signal transduction” should draw more attention as they are the most recent bursts, suggestive of the following hot topics.

## 4 Discussion

### 4.1 Research trends

The concept of phase separation was first introduced into biology in 2009, which was gradually introduced and helped explain many intracellular biological puzzles in cancer research (6, 13, 32, 33). The first study elucidating the biological function of phase separation in cancer was observed in 2016, followed by a slow yearly rise. The publication curve showed that 2018 was the critical point, and since then, the number of related studies exploded. This was most likely because, in 2018, *Science* magazine named phase separation as one of the top 10 scientific accomplishments of the year, which made it more widely known among cancer researchers (34). As more studies about phase separation and cancer were published, the number of citations for those articles increased dramatically, especially in 2022, demonstrating the research’s widespread influence. The average number of citations per article was 24.90, which implied that the publications in this field were generally of a high standard.

### 4.2 Countries and organizations

Regarding a country’s productivity in cancer research, the USA and China are always in the front rank (35–40). Expectedly, in phase separation-related cancer research, the USA and China were the most productive countries among the involved 32 countries, contributing to 75.76% of all publications. However, the AC of papers from China (12.81 times) were the lowest among the top 10 productive countries; this was probably due to the time lag, as the first research from China related to phase separation and cancer was in 2019 (41). Furthermore, the USA and China contributed to five and three institutions among the top 10 active organizations, respectively; Four of these five American institutions ranked in the top five, while those three organizations from China all placed in the bottom five according to the AC. The Howard Hughes Medical Institute from the USA got the most AC, whereas the University of California System from the USA achieved the highest H-index. These results informed us that researchers from the USA possessed both outstanding productivity and excellent influential power in phase separation-associated cancer research.

### 4.3 Journals and authors

Through analysis of the journal distribution, we found that the top 10 productive journals on phase separation in cancer were mainly from three countries, including the USA, England, and Switzerland. The Journal of *Molecular Cell* (IF=19.328) from the USA, with the highest H-index of 9, published most papers (n=10, 3.79%) in this field. *Cell* (IF=66.85), another journal from the USA ranked first by total times of citations, only with five articles, possibly because of its high global impact. *Frontiers in Cell And Developmental Biology* (IF=6.081) and *Molecular Cell* (IF=19.328) published the most papers in the last two years; Nucleic Acids Research (IF=19.16) and Cancers (IF=6.575) were the emerging journals that accepted most of the related documents recently. Researchers could select journals based on our results when submitting future manuscripts associated with phase separation in cancer.

After analysis of the top 10 authors related to phase separation in cancer, it was found that cooperation appears suboptimal between the research groups. Therefore, more collaboration between different groups should be encouraged, which would benefit the future development of this research field. Highly cited papers are regarded as the most authentic and influential achievement in a research field; after listing the top 10 papers regarding phase separation in cancer, it was found that these ten papers accounted for approximately one-third of all citations in this area. Among them, a review entitled “Liquid-Liquid Phase Separation in Disease” was the most cited publication (24). This review described the concept, mechanisms, and features of phase separation and provided a new framework to understand and fight some of the most severe human diseases like cancer.

### 4.4 Keywords and future hotspots

Five clusters were enriched after the keyword co-occurrence analysis. Cluster 1 mainly focused on the role of biomolecular condensates in nuclear homeostasis due to the inclusion of items such as RNA metabolism, nuclear body, epigenetic regulation, p53, SFPQ-NONO, and DNA damage or repair. p53, as a well-known tumor suppressor, could form biomolecular condensates when it is mutated, post-translationally modified, or interacted with its binding partners; the formation of p53 biocondensates could regulate its transcriptional activity, which might change its anticancer properties (42–45). As one cell death inducer, DNA damage can be repaired by SFPQ and NONO, which could accumulate and form membraneless condensates at the DNA damage site (46–48). Cluster 2 represented the biological principle of anticancer drugs for targeting the phase separation process. This cluster contained the keywords of intrinsically disordered regions, transcription, post-translational modification, transcription factor, therapeutics, drug discovery, signal transduction, protein-protein interactions, and cancer metastasis. Proteins possessing intrinsically disordered regions composed of biased amino acids or repetitive sequences are easily post-translationally modified before interacting with each other to form biomolecular condensates (49–51). These membraneless condensates would either act as a signaling platform to activate oncogenic pathways or assemble as transcriptional complexes to promote oncogenic gene expression, both of which could result in carcinogenesis (52–55). Therefore, the discovery of drugs to disrupt multivalent protein-protein interactions would be an excellent choice to disassemble aberrant biocondensates and block tumor signal transduction for anticancer effects (56, 57). Cluster 3 was related to the role of DNA and RNA elements in phase separation-related diseases; it covered the keywords of chromatin, enhancer elements, neurodegenerative diseases, RNA-binding protein, RNA, disease, and gene expression regulation. DNA and RNA, with their binding proteins, could promote the formation of condensates at specific genetic foci, facilitating transcription, chromatin structure maintenance, RNA processing, and other nuclear activities (58). It was demonstrated that dysregulated nuclear condensation was correlated with many human diseases, including cancer and neurodegenerative diseases (59, 60). There were several overlappings between Cluster 3 and Cluster 1; however, research in Cluster 1 mainly focused on the biological function of phase separation in nuclear, while Cluster 3 predominately investigated the pathologic consequences of nuclear condensation disorder. The potential of phase separation in tumor microenvironment-related prognosis and therapy was strengthened in Cluster 4. Tumor microenvironments comprise cancer-associated fibroblasts, immune cells, stromal cells, and noncellular matrix. As the main component of tumor microenvironments, immune-associated cells surveil cancer development by responding to the antigens produced by cancer cells (61, 62). Phase separation has been reported to play a significant role in the immune-relevant signaling assembly during the immune response (63). Therefore, phase separation-regulated immune-relevant signaling pathway is expected to be involved in cancer progression. Specifically, IFN-γ could promote YAP phase separation and cause cancer resistance to anti-PD-1 therapy (64). In addition, the risk models based on the expression of phase separation-related genes were proven to be strongly correlated with tumor immune infiltration and clinicopathological features, which could be applied to predict cancer patients’ prognosis and immunotherapy sensitivity (65–67). The fifth cluster tried to elucidate how cells adopted and addressed internal and external stimuli through phase separation. It was reported that protein mutations or post-translational modifications could affect the secondary or tertiary protein structures, thereby controlling their phase separation behaviors. When internal and external signalings are transduced in protein modifications, cellular activities can be tuned by the dynamics of phase separation formation. For example, during oxidative stress, the oxidization of cysteine 105 and 113 sites on p62 would trigger p62 condensates and subsequently autophagy activation (68); The high level of autophagy would trigger Keap1 autophagic degradation and Nrf2 nuclear translocation, which promotes antioxidant gene transcription to maintain a normal intracellular redox state (69). Undoubtedly, Abnormal autophagy regulation is regarded as another causative factor for cancer (70, 71).

For the perspectives of phase separation in cancer research, the keywords “tumor microenvironment”, “immunotherapy”, “prognosis”, “p53”, “cell death”, “intrinsically disordered region”, “immune response”, and “signal transduction” are likely to gain more attention in the future as our keyword co-occurrence and burst analysis showed that they were recent research hotspots. The relationship between aberrant phase separation and cancer has been well documented; discovering more phase separation-related oncogenes and related transduction molecules facilitates precise cancer prognosis and helps design new pharmaceuticals to kill cancer cells. In addition to intrinsic self-causing mechanisms, abnormal biocondensations are also determined by crosstalks between the tumor microenvironments and cancer cells; however, how intrinsically disordered region-possessing proteins such as p53 initiate phase separation to drive oncogenesis under various extracellular stimuli conditions remains ambiguous; as the main component of tumor microenvironments, immune cells such as T cells and NK cells often loss their anticancer effects during cancer progression, therefore, recovering their immune surveillance capacity would benefit anticancer therapies. Phase separation is required to activate key immune signalings such as T cell receptor (TCR) and NF-κB pathways in immune cells. Therefore, it is interesting to investigate whether targeting phase separation could be used in T cell or NK cell engineering that leads to the establishment of new immunotherapy approaches (72, 73). Collectively, future phase separation-related cancer research should not only focus on cancer cells, but also expand to tumor microenvironments, including cancer-associated fibroblasts, immune cells, stromal cells, and noncellular matrix; Exploring the interactive influence between phase separation and tumor microenvironments (like immune cells), and defining the impacts of these interrelations on cancer behavior might shed light on a more precise and efficient approach to cancer prognosis and therapeutics.

### 4.5 Limitations

The bibliometric analysis allows for a more comprehensive and intuitive picture of the current state of research. In contrast to traditional reviews, our study would provide objective insights into the future landscapes of phase separation-related cancer studies. However, several limitations are not inevitable. First, although the WoSCC database had a high article coverage, insufficient data collection and variable results might appear due to the inability to obtain all papers from multiple databases. Second, the bibliometric data will evolve over time, which leads to inconsistent results and requires prompt updating. Additionally, there are some restrictions on the literature type when screening literature. For example, some essential non-English studies, conference abstracts, and editorials are excluded from our research. Finally, the bibliometric analysis is unable to depict the whole picture of the current research status. For instance, some high-quality articles were not given sufficient attention due to time lags, which is the possibility of missed research direction.

### 4.6 Conclusion

Our results showed that phase separation-related cancer research remained in the hot streak period and exhibited a promising outlook. The USA was the leading and most influential country. Although inter-agency collaboration existed, cooperation among research groups was rare, and no author dominated this field at the current stage. Investigating the interfaced effects between phase separation and tumor microenvironments on carcinoma behaviors, and constructing relevant prognoses and therapeutics such as immune infiltration-based prognosis and immunotherapy might be the next research trend in the study of phase separation and cancer.

## Data availability statement

The original contributions presented in the study are included in the article/**Supplementary Material**. Further inquiries can be directed to the corresponding author.

## Author contributions

This study was conceptualized and designed by DW and JZ. ML and DW collected and analyzed the database. ML, YZ and DW interpreted the results. ML, YZ and DW drafted and approved the final version of the manuscripts. All authors contributed to the article and approved the submitted version.

## References

[1] Hanahan D Weinberg RA. Hallmarks of cancer: the next generation. Cell (2011) 144(5):646–74. doi: 10.1016/j.cell.2011.02.013 21376230

[2] Hanahan D. Hallmarks of cancer: new dimensions. Cancer Discov (2022) 12(1):31–46. doi: 10.1158/2159-8290.CD-21-1059 35022204

[3] Medicine Io. Cancer and the environment: gene-environment interaction. Wilson S Jones L Coussens C Hanna K, editors. Washington, DC: The National Academies Press (2002). p. 160. doi: 10.17226/10464 25057619

[4] Aguzzi A Altmeyer M. Phase separation: linking cellular compartmentalization to disease. Trends Cell Biol (2016) 26(7):547–58. doi: 10.1016/j.tcb.2016.03.004 27051975

[5] Wang W Chen Y Xu A Cai M Cao J Zhu H. Protein phase separation: a novel therapy for cancer? Br J Pharmacol (2020) 177(22):5008–30. doi: 10.1111/bph.15242 PMC7589017 32851637

[6] Taniue K Akimitsu N. Aberrant phase separation and cancer. FEBS J (2022) 289(1):17–39. doi: 10.1111/febs.15765 33583140

[7] Mitrea DM Kriwacki RW. Phase separation in biology; functional organization of a higher order. Cell Commun Signal (2016) 14:1. doi: 10.1186/s12964-015-0125-7 26727894 PMC4700675

[8] Banani SF Lee HO Hyman AA Rosen MK. Biomolecular condensates: organizers of cellular biochemistry. Nat Rev Mol Cell Biol (2017) 18(5):285–98. doi: 10.1038/nrm.2017.7 PMC7434221 28225081

[9] Lafontaine DLJ Riback JA Bascetin R Brangwynne CP. The nucleolus as a multiphase liquid condensate. Nat Rev Mol Cell Biol (2020) 22(3):165–82. doi: 10.1038/s41580-020-0272-6 32873929

[10] Boija A Klein IA Young RA. Biomolecular condensates and cancer. Cancer Cell (2021) 39(2):174–92. doi: 10.1016/j.ccell.2020.12.003 PMC8721577 33417833

[11] Fare CM Villani A Drake LE Shorter J. Higher-order organization of biomolecular condensates. Open Biol (2021) 11(6):210137. doi: 10.1098/rsob.210137 34129784 PMC8205532

[12] Strulson CA Molden RC Keating CD Bevilacqua PC. Rna catalysis through compartmentalization. Nat Chem (2012) 4(11):941–6. doi: 10.1038/nchem.1466 23089870

[13] Mehta S Zhang J. Liquid-liquid phase separation drives cellular function and dysfunction in cancer. Nat Rev Cancer (2022) 22(4):239–52. doi: 10.1038/s41568-022-00444-7 PMC10036213 35149762

[14] Tong X Tang R Xu J Wang W Zhao Y Yu X. Liquid-liquid phase separation in tumor biology. Signal Transduct Target Ther (2022) 7(1):221. doi: 10.1038/s41392-022-01076-x 35803926 PMC9270353

[15] Li W Hu J Shi B Palomba F Digman MA Gratton E. Biophysical properties of Akap95 protein condensates regulate splicing and tumorigenesis. Nat Cell Biol (2020) 22(8):960–72. doi: 10.1038/s41556-020-0550-8 PMC7425812 32719551

[16] Ma S Lu CC Yang LY Wang JJ Wang BS Cai HQ. Anxa2 promotes esophageal cancer progression by activating myc-Hif1a-Vegf axis. J Exp Clin Cancer Res (2018) 37(1):183. doi: 10.1186/s13046-018-0851-y 30081903 PMC6091180

[17] Song MS Grabocka E. Stress granules in cancer. Rev Physiol Biochem Pharmacol (2023) 185:25–52. doi: 10.1007/112_2020_37 32789791 PMC8786373

[18] Zhu G Xie J Kong W Xie J Li Y Du L. Phase separation of disease-associated Shp2 mutants underlies mapk hyperactivation. Cell (2020) 183(2):490–502 e18. doi: 10.1016/j.cell.2020.09.002 33002410 PMC7572904

[19] Igelmann S Lessard F Ferbeyre G. Liquid-liquid phase separation in cancer signaling, metabolism and anticancer therapy. Cancers (Basel) (2022) 14(7):1830. doi: 10.3390/cancers14071830 35406602 PMC8997759

[20] Zhu G Xie J Fu Z Wang M Zhang Q He H. Pharmacological inhibition of src-1 phase separation suppresses yap oncogenic transcription activity. Cell Res (2021) 31(9):1028–31. doi: 10.1038/s41422-021-00504-x PMC8410775 33850322

[21] Xie J He H Kong W Li Z Gao Z Xie D. Targeting androgen receptor phase separation to overcome antiandrogen resistance. Nat Chem Biol (2022) 18(12):1341–50. doi: 10.1038/s41589-022-01151-y 36229685

[22] Donthu N Kumar S Mukherjee D Pandey N Lim WM. How to conduct a bibliometric analysis: an overview and guidelines. J Business Res (2021) 133:285–96. doi: 10.1016/j.jbusres.2021.04.070

[23] Brangwynne CP Eckmann CR Courson DS Rybarska A Hoege C Gharakhani J. Germline p granules are liquid droplets that localize by controlled Dissolution/Condensation. Science (2009) 324(5935):1729–32. doi: 10.1126/science.1172046 19460965

[24] Alberti S Dormann D. Liquid-liquid phase separation in disease. Annu Rev Genet (2019) 53:171–94. doi: 10.1146/annurev-genet-112618-043527 31430179

[25] Guccione E Richard S. The regulation, functions and clinical relevance of arginine methylation. Nat Rev Mol Cell Biol (2019) 20(10):642–57. doi: 10.1038/s41580-019-0155-x 31350521

[26] Stadhouders R Filion GJ Graf T. Transcription factors and 3d genome conformation in cell-fate decisions. Nature (2019) 569(7756):345–54. doi: 10.1038/s41586-019-1182-7 31092938

[27] Bouchard JJ Otero JH Scott DC Szulc E Martin EW Sabri N. Cancer mutations of the tumor suppressor spop disrupt the formation of active, phase-separated compartments. Mol Cell (2018) 72(1):19–36 e8. doi: 10.1016/j.molcel.2018.08.027 30244836 PMC6179159

[28] Nair SJ Yang L Meluzzi D Oh S Yang F Friedman MJ. Phase separation of ligand-activated enhancers licenses cooperative chromosomal enhancer assembly. Nat Struct Mol Biol (2019) 26(3):193–203. doi: 10.1038/s41594-019-0190-5 30833784 PMC6709854

[29] Sanchez-Martin P Komatsu M. P62/Sqstm1 - steering the cell through health and disease. J Cell Sci (2018) 131(21):jcs222836. doi: 10.1242/jcs.222836 30397181

[30] Klein IA Boija A Afeyan LK Hawken SW Fan M Dall'Agnese A. Partitioning of cancer therapeutics in nuclear condensates. Science (2020) 368(6497):1386–92. doi: 10.1126/science.aaz4427 PMC7735713 32554597

[31] Isoda T Moore AJ He Z Chandra V Aida M Denholtz M. Non-coding transcription instructs chromatin folding and compartmentalization to dictate enhancer-promoter communication and T cell fate. Cell (2017) 171(1):103–19 e18. doi: 10.1016/j.cell.2017.09.001 28938112 PMC5621651

[32] Jiang S Fagman JB Chen C Alberti S Liu B. Protein phase separation and its role in tumorigenesis. Elife (2020) 9:e60264. doi: 10.7554/eLife.60264 33138914 PMC7609067

[33] Peng Q Tan S Xia L Wu N Oyang L Tang Y. Phase separation in cancer: from the impacts and mechanisms to treatment potentials. Int J Biol Sci (2022) 18(13):5103–22. doi: 10.7150/ijbs.75410 PMC9379413 35982902

[34] Hand E Vogel G Garber K Kaiser J Servick K Clery D. Runners-up. Science (2018) 362(6421):1346–51. doi: 10.1126/science.362.6421.1346 30573611

[35] Teles RHG Moralles HF Cominetti MR. Global trends in nanomedicine research on triple negative breast cancer: a bibliometric analysis. Int J Nanomedicine (2018) 13:2321–36. doi: 10.2147/IJN.S164355 PMC5910795 29713164

[36] Da W Tao Z Meng Y Wen K Zhou S Yang K. A 10-year bibliometric analysis of osteosarcoma and cure from 2010 to 2019. BMC Cancer (2021) 21(1):115. doi: 10.1186/s12885-021-07818-4 33541299 PMC7863524

[37] Shi Y Wei W Li L Wei Q Jiang F Xia G. The global status of research in breast cancer liver metastasis: a bibliometric and visualized analysis. Bioengineered (2021) 12(2):12246–62. doi: 10.1080/21655979.2021.2006552 PMC8810156 34783637

[38] Ma L Ma J Teng M Li Y. Visual analysis of colorectal cancer immunotherapy: a bibliometric analysis from 2012 to 2021. Front Immunol (2022) 13:843106. doi: 10.3389/fimmu.2022.843106 35432385 PMC9009266

[39] Shen J Shen H Ke L Chen J Dang X Liu B. Knowledge mapping of immunotherapy for hepatocellular carcinoma: a bibliometric study. Front Immunol (2022) 13:815575. doi: 10.3389/fimmu.2022.815575 35173728 PMC8841606

[40] Zyoud SH Al-Jabi SW Amer R Shakhshir M Shahwan M Jairoun AA. Global research trends on the links between the gut microbiome and cancer: a visualization analysis. J Transl Med (2022) 20(1):83. doi: 10.1186/s12967-022-03293-y 35148757 PMC8832721

[41] Ming Y Chen X Xu Y Wu Y Wang C Zhang T. Targeting liquid-liquid phase separation in pancreatic cancer. Transl Cancer Res (2019) 8(1):96–103. doi: 10.21037/tcr.2019.01.06 35116738 PMC8798987

[42] Chen C Fu G Guo Q Xue S Luo SZ. Phase separation of P53 induced by its unstructured basic region and prevented by oncogenic mutations in tetramerization domain. Int J Biol Macromol (2022) 222(Pt A):207–16. doi: 10.1016/j.ijbiomac.2022.09.087 36108750

[43] Dai Z Li G Chen Q Yang X. Ser392 phosphorylation modulated a switch between P53 and transcriptional condensates. Biochim Biophys Acta (BBA) - Gene Regul Mech (2022) 1865(4):194827. doi: 10.1016/j.bbagrm.2022.194827 35618207

[44] Marques MA de Oliveira GAP Silva JL. The chameleonic behavior of P53 in health and disease: the transition from a client to an aberrant condensate scaffold in cancer. Essays Biochem (2022) 66(7):1023–33. doi: 10.1042/EBC20220064 36350030

[45] Liebl MC Hofmann TG. Regulating the P53 tumor suppressor network at pml biomolecular condensates. Cancers (Basel) (2022) 14(19):4549. doi: 10.3390/cancers14194549 36230470 PMC9558958

[46] Fan XJ Wang YL Zhao WW Bai SM Ma Y Yin XK. Nono phase separation enhances DNA damage repair by accelerating nuclear egfr-induced DNA-Pk activation. Am J Cancer Res (2021) 11(6):2838–52. PMC8263645 34249431

[47] Fijen C Rothenberg E. The evolving complexity of DNA damage foci: rna, condensates and chromatin in DNA double-strand break repair. DNA Repair (Amst) (2021) 105:103170. doi: 10.1016/j.dnarep.2021.103170 34256335 PMC8364513

[48] Sanchez A Lee D Kim DI Miller KM. Making connections: integrative signaling mechanisms coordinate DNA break repair in chromatin. Front Genet (2021) 12:747734. doi: 10.3389/fgene.2021.747734 34659365 PMC8514019

[49] Kato M Han TW Xie S Shi K Du X Wu LC. Cell-free formation of rna granules: low complexity sequence domains form dynamic fibers within hydrogels. Cell (2012) 149(4):753–67. doi: 10.1016/j.cell.2012.04.017 PMC6347373 22579281

[50] Molliex A Temirov J Lee J Coughlin M Kanagaraj AP Kim HJ. Phase separation by low complexity domains promotes stress granule assembly and drives pathological fibrillization. Cell (2015) 163(1):123–33. doi: 10.1016/j.cell.2015.09.015 PMC5149108 26406374

[51] Li J Zhang M Ma W Yang B Lu H Zhou F. Post-translational modifications in liquid-liquid phase separation: a comprehensive review. Mol BioMed (2022) 3(1):13. doi: 10.1186/s43556-022-00075-2 35543798 PMC9092326

[52] Peng L Li EM Xu LY. From start to end: phase separation and transcriptional regulation. Biochim Biophys Acta Gene Regul Mech (2020) 1863(12):194641. doi: 10.1016/j.bbagrm.2020.194641 33017669

[53] Tulpule A Guan J Neel DS Allegakoen HR Lin YP Brown D. Kinase-mediated ras signaling Via membraneless cytoplasmic protein granules. Cell (2021) 184(10):2649–64 e18. doi: 10.1016/j.cell.2021.03.031 33848463 PMC8127962

[54] Hu X Wu X Berry K Zhao C Xin D Ogurek S. Nuclear condensates of yap fusion proteins alter transcription to drive ependymoma tumourigenesis. Nat Cell Biol (2023) 25(2):323–36. doi: 10.1038/s41556-022-01069-6 36732631

[55] Liu X Ye Y Zhu L Xiao X Zhou B Gu Y. Niche stiffness sustains cancer stemness Via taz and nanog phase separation. Nat Commun (2023) 14(1):238. doi: 10.1038/s41467-023-35856-y 36646707 PMC9842735

[56] Martinez MS. Intrinsically disordered proteins as drug targets. MOJ Proteomics Bioinf (2017) 5(2):69–73. doi: 10.15406/mojpb.2017.05.00157

[57] Santofimia-Castano P Rizzuti B Xia Y Abian O Peng L Velazquez-Campoy A. Targeting intrinsically disordered proteins involved in cancer. Cell Mol Life Sci (2020) 77(9):1695–707. doi: 10.1007/s00018-019-03347-3 PMC7190594 31667555

[58] Sabari BR Dall'Agnese A Young RA. Biomolecular condensates in the nucleus. Trends Biochem Sci (2020) 45(11):961–77. doi: 10.1016/j.tibs.2020.06.007 PMC7572565 32684431

[59] Zbinden A Perez-Berlanga M De Rossi P Polymenidou M. Phase separation and neurodegenerative diseases: a disturbance in the force. Dev Cell (2020) 55(1):45–68. doi: 10.1016/j.devcel.2020.09.014 33049211

[60] Ito S Das ND Umehara T Koseki H. Factors and mechanisms that influence chromatin-mediated enhancer-promoter interactions and transcriptional regulation. Cancers (Basel) (2022) 14(21):5404. doi: 10.3390/cancers14215404 36358822 PMC9659172

[61] Chew V Toh HC Abastado JP. Immune microenvironment in tumor progression: characteristics and challenges for therapy. J Oncol (2012) 2012:608406. doi: 10.1155/2012/608406 22927846 PMC3423944

[62] Hiam-Galvez KJ Allen BM Spitzer MH. Systemic immunity in cancer. Nat Rev Cancer (2021) 21(6):345–59. doi: 10.1038/s41568-021-00347-z PMC8034277 33837297

[63] Xiao Q McAtee CK Su X. Phase separation in immune signalling. Nat Rev Immunol (2022) 22(3):188–99. doi: 10.1038/s41577-021-00572-5 PMC9674404 34230650

[64] Yu M Peng Z Qin M Liu Y Wang J Zhang C. Interferon-gamma induces tumor resistance to anti-Pd-1 immunotherapy by promoting yap phase separation. Mol Cell (2021) 81(6):1216–30 e9. doi: 10.1016/j.molcel.2021.01.010 33606996

[65] Zhang Y Li J Feng D Peng X Wang B Han T. Systematic analysis of molecular characterization and clinical relevance of liquid-liquid phase separation regulators in digestive system neoplasms. Front Cell Dev Biol (2021) 9:820174. doi: 10.3389/fcell.2021.820174 35252219 PMC8891544

[66] Sun L Liu XP Yan X Wu S Tang X Chen C. Identification of molecular subtypes based on liquid-liquid phase separation and cross-talk with immunological phenotype in bladder cancer. Front Immunol (2022) 13:1059568. doi: 10.3389/fimmu.2022.1059568 36518754 PMC9742536

[67] Wang J Meng F Mao F. Single cell sequencing analysis and transcriptome analysis constructed the liquid-liquid phase Separation(Llps)-related prognostic model for endometrial cancer. Front Oncol (2022) 12:1005472. doi: 10.3389/fonc.2022.1005472 36185238 PMC9515536

[68] Carroll B Otten EG Manni D Stefanatos R Menzies FM Smith GR. Oxidation of Sqstm1/P62 mediates the link between redox state and protein homeostasis. Nat Commun (2018) 9(1):256. doi: 10.1038/s41467-017-02746-z 29343728 PMC5772351

[69] Taguchi K Fujikawa N Komatsu M Ishii T Unno M Akaike T. Keap1 degradation by autophagy for the maintenance of redox homeostasis. Proc Natl Acad Sci USA (2012) 109(34):13561–6. doi: 10.1073/pnas.1121572109 PMC3427110 22872865

[70] Yun CW Lee SH. The roles of autophagy in cancer. Int J Mol Sci (2018) 19(11):3466. doi: 10.3390/ijms19113466 30400561 PMC6274804

[71] Chen C Gao H Su X. Autophagy-related signaling pathways are involved in cancer (Review). Exp Ther Med (2021) 22(1):710. doi: 10.3892/etm.2021.10142 34007319 PMC8120650

[72] Lei X Lei Y Li JK Du WX Li RG Yang J. Immune cells within the tumor microenvironment: biological functions and roles in cancer immunotherapy. Cancer Lett (2020) 470:126–33. doi: 10.1016/j.canlet.2019.11.009 31730903

[73] Binnewies M Roberts EW Kersten K Chan V Fearon DF Merad M. Understanding the tumor immune microenvironment (Time) for effective therapy. Nat Med (2018) 24(5):541–50. doi: 10.1038/s41591-018-0014-x PMC5998822 29686425

