# Supplementary material for: The global landscape and research trend of phase separation in cancer: a bibliometric analysis and visualization
Source: Front Oncol. 2023 Jun 2;13:1170157. doi: 10.3389/fonc.2023.1170157 (PMC10272442; doi:10.3389/fonc.2023.1170157)
Supplement: Supplementary file 1 [file DataSheet_1.docx]

Supplementary Material

The global landscape and research trend of phase separation in cancer: A bibliometric analysis and visualization

Mengzhu Li^1,2,3,4,5†^, Yizhan, Zhang^1,2,3,4,5†^, JiaJun Zhao^2,3,4,5^, Dawei Wang^1,2,3,4,5*^

^1^Department of Endocrinology, Shandong Provincial Hospital, Shandong University, Jinan, China.

^2^Department of Endocrinology, Shandong Provincial Hospital Affiliated to Shandong First Medical University, Jinan, China.

^3^Shandong Key Laboratory of Endocrinology and Lipid Metabolism, Jinan, China.

^4^Shandong Institute of Endocrine and Metabolic Diseases, Jinan, China.

^5^Key Laboratory of Endocrine Glucose & Lipids Metabolism and Brain Aging (Shandong First Medical University), Ministry of Education, Jinan, China.

† **Equal contribution:**

Mengzhu Li^1,2,3,4,5†^ and Yizhan, Zhang^1,2,3,4,5†^ contributed equally to this work and shared the first authorship.

*** Correspondence:**Dawei Wang
[wangdawei@sdfmu.edu.cn](mailto:wangdawei@sdfmu.edu.cn)

**The selected 264 papers for deep analysis:**

Aguzzi, A., & Altmeyer, M. (2016). Phase Separation: Linking Cellular Compartmentalization to Disease. *Trends in Cell Biology, 26*(7), 547-558. doi:10.1016/j.tcb.2016.03.004

Valentin-Vega, Y. A., Wang, Y. D., Parker, M., Patmore, D. M., Kanagaraj, A., Moore, J., . . . Taylor, J. P. (2016). Cancer-associated DDX3X mutations drive stress granule assembly and impair global translation. *Scientific Reports, 6*, 16. doi:10.1038/srep25996

Isoda, T., Moore, A. J., He, Z. R., Chandra, V., Aida, M., Denholtz, M., . . . Murre, C. (2017). Non-coding Transcription Instructs Chromatin Folding and Compartmentalization to Dictate Enhancer-Promoter Communication and T Cell Fate. *Cell, 171*(1), 103-+. doi:10.1016/j.cell.2017.09.001

Jin, M. Y., Fuller, G. G., Han, T., Yao, Y., Alessi, A. F., Freeberg, M. A., . . . Kim, J. K. (2017). Glycolytic Enzymes Coalesce in G Bodies under Hypoxic Stress. *Cell Reports, 20*(4), 895-908. doi:10.1016/j.celrep.2017.06.082

Sawyer, I. A., Hager, G. L., & Dundr, M. (2017). Specific genomic cues regulate Cajal body assembly. *Rna Biology, 14*(6), 791-803. doi:10.1080/15476286.2016.1243648

Stanek, D., & Fox, A. H. (2017). Nuclear bodies: news insights into structure and function. *Current Opinion in Cell Biology, 46*, 94-101. doi:10.1016/j.ceb.2017.05.001

Boeynaems, S., Tompa, P., & Van den Bosch, L. (2018). Phasing in on the cell cycle. *Cell Division, 13*, 8. doi:10.1186/s13008-018-0034-4

Bouchard, J. J., Otero, J. H., Scott, D. C., Szulc, E., Martin, E. W., Sabri, N., . . . Mittag, T. (2018). Cancer Mutations of the Tumor Suppressor SPOP Disrupt the Formation of Active, Phase-Separated Compartments. *Molecular Cell, 72*(1), 19-+. doi:10.1016/j.molcel.2018.08.027

Cermakova, K., & Hodges, H. C. (2018). Next-Generation Drugs and Probes for Chromatin Biology: From Targeted Protein Degradation to Phase Separation. *Molecules, 23*(8), 26. doi:10.3390/molecules23081958

Cloer, E. W., Siesser, P. F., Cousins, E. M., Goldfar, D., Mowrey, D. D., Harrison, J. S., . . . Major, M. B. (2018). p62-Dependent Phase Separation of Patient-Derived KEAP1 Mutations and NRF2. *Molecular and Cellular Biology, 38*(22), 26. doi:10.1128/mcb.00644-17

Prouteau, M., & Loewith, R. (2018). Regulation of Cellular Metabolism through Phase Separation of Enzymes. *Biomolecules, 8*(4), 14. doi:10.3390/biom8040160

Sanchez-Martin, P., & Komatsu, M. (2018). p62/SQSTM1-steering the cell through health and disease. *Journal of Cell Science, 131*(21), 13. doi:10.1242/jcs.222836

Alberti, S., & Dormann, D. (2019). Liquid-Liquid Phase Separation in Disease. In N. M. Bonini (Ed.), *Annual Review of Genetics, Vol 53* (Vol. 53, pp. 171-+). Palo Alto: Annual Reviews.

Correll, C. C., Bartek, J., & Dundr, M. (2019). The Nucleolus: A Multiphase Condensate Balancing Ribosome Synthesis and Translational Capacity in Health, Aging and Ribosomopathies. *Cells, 8*(8), 19. doi:10.3390/cells8080869

Cuneo, M. J., & Mittag, T. (2019). The ubiquitin ligase adaptor SPOP in cancer. *Febs Journal, 286*(20), 3946-3958. doi:10.1111/febs.15056

Darling, A. L., Zaslavsky, B. Y., & Uversky, V. N. (2019). Intrinsic Disorder-Based Emergence in Cellular Biology: Physiological and Pathological Liquid-Liquid Phase Transitions in Cells. *Polymers, 11*(6), 23. doi:10.3390/polym11060990

Fant, C. B., & Taatjes, D. J. (2019). Regulatory functions of the Mediator kinases CDK8 and CDK19. *Transcription-Austin, 10*(2), 76-90. doi:10.1080/21541264.2018.1556915

Guccione, E., & Richard, S. (2019). The regulation, functions and clinical relevance of arginine methylation. *Nature Reviews Molecular Cell Biology, 20*(10), 642-657. doi:10.1038/s41580-019-0155-x

Hall, A. C., Ostrowski, L. A., & Mekhail, K. (2019). Phase Separation as a Melting Pot for DNA Repeats. *Trends in Genetics, 35*(8), 589-600. doi:10.1016/j.tig.2019.05.001

Latonen, L. (2019). Phase-to-Phase With Nucleoli - Stress Responses, Protein Aggregation and Novel Roles of RNA. *Frontiers in Cellular Neuroscience, 13*, 10. doi:10.3389/fncel.2019.00151

Min, J., Wright, W. E., & Shay, J. W. (2019). Clustered telomeres in phase-separated nuclear condensates engage mitotic DNA synthesis through BLM and RAD52. *Genes & Development, 33*(13-14), 814-827. doi:10.1101/gad.324905.119

Min, Y., Chen, X., Xu, Y. P., Wu, Y. Y., Wang, C. L., Zhang, T. L., . . . Fan, Y. H. (2019). Targeting liquid-liquid phase separation in pancreatic cancer. *Translational Cancer Research, 8*(1), 96-103. doi:10.21037/tcr.2019.01.06

Nair, S. J., Yang, L., Meluzzi, D., Oh, S., Yang, F., Friedman, M. J., . . . Rosenfeld, M. G. (2019). Phase separation of ligand-activated enhancers licenses cooperative chromosomal enhancer assembly. *Nature Structural & Molecular Biology, 26*(3), 193-+. doi:10.1038/s41594-019-0190-5

Park, J. E., Zhang, L., Bang, J. K., Andresson, T., DiMaio, F., & Lee, K. S. (2019). Phase separation of Polo-like kinase 4 by autoactivation and clustering drives centriole biogenesis. *Nature Communications, 10*, 19. doi:10.1038/s41467-019-12619-2

Pirogov, S. A., Gvozdev, V. A., & Klenov, M. S. (2019). Long Noncoding RNAs and Stress Response in the Nucleolus. *Cells, 8*(7), 16. doi:10.3390/cells8070668

Rubio, K., Dobersch, S., & Barreto, G. (2019). Functional interactions between scaffold proteins, noncoding RNAs, and genome loci induce liquid-liquid phase separation as organizing principle for 3-dimensional nuclear architecture: implications in cancer. *Faseb Journal, 33*(5), 5814-5822. doi:10.1096/fj.201802715R

Ryan, J. J., Sprunger, M. L., Holthaus, K., Shorter, J., & Jackrel, M. E. (2019). Engineered protein disaggregases mitigate toxicity of aberrant prion-like fusion proteins underlying sarcoma. *Journal of Biological Chemistry, 294*(29), 11286-11296. doi:10.1074/jbc.RA119.009494

Safari, M. S., Wang, Z. Q., Tailor, K., Kolomeisky, A. B., Conrad, J. C., & Vekilov, P. G. (2019). Anomalous Dense Liquid Condensates Host the Nucleation of Tumor Suppressor p53 Fibrils. *Iscience, 12*, 342-+. doi:10.1016/j.isci.2019.01.027

Sawyer, I. A., Bartek, J., & Dundr, M. (2019). Phase separated microenvironments inside the cell nucleus are linked to disease and regulate epigenetic state, transcription and RNA processing. *Seminars in Cell & Developmental Biology, 90*, 94-103. doi:10.1016/j.semcdb.2018.07.001

Schaefer, K. N., & Peifer, M. (2019). Wnt/Beta-Catenin Signaling Regulation and a Role for Biomolecular Condensates. *Developmental Cell, 48*(4), 429-444. doi:10.1016/j.devcel.2019.01.025

Spannl, S., Tereshchenko, M., Mastromarco, G. J., Ihn, S. J., & Lee, H. O. (2019). Biomolecular condensates in neurodegeneration and cancer. *Traffic, 20*(12), 890-911. doi:10.1111/tra.12704

Stadhouders, R., Filion, G. J., & Graf, T. (2019). Transcription factors and 3D genome conformation in cell-fate decisions. *Nature, 569*(7756), 345-354. doi:10.1038/s41586-019-1182-7

Thandapani, P. (2019). Super-enhancers in cancer. *Pharmacology & Therapeutics, 199*, 129-138. doi:10.1016/j.pharmthera.2019.02.014

Verdile, V., De Paola, E., & Paronetto, M. P. (2019). Aberrant Phase Transitions: Side Effects and Novel Therapeutic Strategies in Human Disease. *Frontiers in Genetics, 10*, 14. doi:10.3389/fgene.2019.00173

Wang, X., Cairns, M. J., & Yan, J. (2019). Super-enhancers in transcriptional regulation and genome organization. *Nucleic Acids Research, 47*(22), 11481-11496. doi:10.1093/nar/gkz1038

Beato, M., & Sharma, P. (2020). Peptidyl Arginine Deiminase 2 (PADI2)-Mediated Arginine Citrullination Modulates Transcription in Cancer. *International Journal of Molecular Sciences, 21*(4), 16. doi:10.3390/ijms21041351

Beato, M., Wright, R. H. G., & Le Dily, F. (2020). 90 YEARS OF PROGESTERONE Molecular mechanisms of progesterone receptor action on the breast cancer genome. *Journal of Molecular Endocrinology, 65*(1), T65-T79. doi:10.1530/jme-19-0266

Beaulieu, M. E., Castillo, F., & Soucek, L. (2020). Structural and Biophysical Insights into the Function of the Intrinsically Disordered Myc Oncoprotein. *Cells, 9*(4), 27. doi:10.3390/cells9041038

Becskei, A. (2020). Tuning up Transcription Factors for Therapy. *Molecules, 25*(8), 19. doi:10.3390/molecules25081902

Belardi, B., Son, S. M., Felce, J. H., Dustin, M. L., & Fletcher, D. A. (2020). Cell-cell interfaces as specialized compartments directing cell function. *Nature Reviews Molecular Cell Biology, 21*(12), 750-764. doi:10.1038/s41580-020-00298-7

Chen, X. R., Ma, Q. W., Shang, Z. Q., & Niu, Y. J. (2020). Super-enhancer in prostate cancer: transcriptional disorders and therapeutic targets. *Npj Precision Oncology, 4*(1), 7. doi:10.1038/s41698-020-00137-0

Corless, S., Hocker, S., & Erhardt, S. (2020). Centromeric RNA and Its Function at and Beyond Centromeric Chromatin. *Journal of Molecular Biology, 432*(15), 4257-4269. doi:10.1016/j.jmb.2020.03.027

de Oliveira, G. A. P., Cordeiro, Y., Silva, J. L., & Vieira, T. (2020). Liquid-liquid phase transitions and amyloid aggregation in proteins related to cancer and neurodegenerative diseases. In R. Donev (Ed.), *Protein Misfolding* (Vol. 118, pp. 289-331). London: Academic Press Ltd-Elsevier Science Ltd.

Drier, Y. (2020). Enhancer and superenhancer regulation and its disruption in cancer. *Current Opinion in Systems Biology, 19*, 24-30. doi:10.1016/j.coisb.2020.05.002

Feng, Y. L., & Pauklin, S. (2020). Revisiting 3D chromatin architecture in cancer development and progression. *Nucleic Acids Research, 48*(19), 10632-10647. doi:10.1093/nar/gkaa747

Gibbs, E., Perrone, B., Hassan, A., Kummerle, R., & Kriwacki, R. (2020). NPM1 exhibits structural and dynamic heterogeneity upon phase separation with the p14ARF tumor suppressor. *Journal of Magnetic Resonance, 310*, 8. doi:10.1016/j.jmr.2019.106646

Han, X. Y., Yu, D., Gu, R. R., Jia, Y. J., Wang, Q., Jaganathan, A., . . . Zeng, L. (2020). Roles of the BRD4 short isoform in phase separation and active gene transcription. *Nature Structural & Molecular Biology, 27*(4), 333-+. doi:10.1038/s41594-020-0394-8

Jiang, S., Fagman, J. B., Chen, C. Y., Alberti, S., & Liu, B. D. (2020). Protein phase separation and its role in tumorigenesis. *Elife, 9*, 27. doi:10.7554/eLife.60264

Kamagata, K., Kanbayashi, S., Honda, M., Itoh, Y., Takahashi, H., Kameda, T., . . . Takahashi, S. (2020). Liquid-like droplet formation by tumor suppressor p53 induced by multivalent electrostatic interactions between two disordered domains. *Scientific Reports, 10*(1), 12. doi:10.1038/s41598-020-57521-w

Kantidze, O. L., Gurova, K. V., Studitsky, V. M., & Razin, S. V. (2020). The 3D Genome as a Target for Anticancer Therapy. *Trends in Molecular Medicine, 26*(2), 141-149. doi:10.1016/j.molmed.2019.09.011

Klein, I. A., Boija, A., Afeyan, L. K., Hawken, S. W., Fan, M. Y., Dall'Agnese, A., . . . Young, R. A. (2020). Partitioning of cancer therapeutics in nuclear condensates. *Science, 368*(6497), 1386-+. doi:10.1126/science.aaz4427

Lee, A. K., Klein, J., Tacer, K. F., Lord, T., Oatley, M. J., Oatley, J. M., . . . Potts, P. R. (2020). Translational Repression of G3BP in Cancer and Germ Cells Suppresses Stress Granules and Enhances Stress Tolerance. *Molecular Cell, 79*(4), 645-+. doi:10.1016/j.molcel.2020.06.037

Lemos, C., Schulze, L., Weiske, J., Meyer, H., Braeuer, N., Barak, N., . . . Steigemann, P. (2020). Identification of Small Molecules that Modulate Mutant p53 Condensation. *Iscience, 23*(9), 27. doi:10.1016/j.isci.2020.101517

Leung, A. K. L. (2020). Poly(ADP-ribose): A Dynamic Trigger for Biomolecular Condensate Formation. *Trends in Cell Biology, 30*(5), 370-383. doi:10.1016/j.tcb.2020.02.002

Li, T. M., Ren, J., Husmann, D., Coan, J. P., Gozani, O., & Chua, K. F. (2020). Multivalent tumor suppressor adenomatous polyposis coli promotes Axin biomolecular condensate formation and efficient beta-catenin degradation. *Scientific Reports, 10*(1), 9. doi:10.1038/s41598-020-74080-2

Li, W., Hu, J., Shi, B., Palomba, F., Digman, M. A., Gratton, E., & Jiang, H. (2020). Biophysical properties of AKAP95 protein condensates regulate splicing and tumorigenesis. *Nature Cell Biology, 22*(8), 960-+. doi:10.1038/s41556-020-0550-8

Li, W., & Jiang, H. (2020). Regulation of tumorigenic splicing by protein condensates with specific biophysical properties. *Molecular & Cellular Oncology, 7*(6), 2. doi:10.1080/23723556.2020.1819753

Lu, Y., Wu, T. T., Gutman, O., Lu, H. S., Zhou, Q., Henis, Y. I., & Luo, K. X. (2020). Phase separation of TAZ compartmentalizes the transcription machinery to promote gene expression. *Nature Cell Biology, 22*(4), 26. doi:10.1038/s41556-020-0485-0

Michmerhuizen, N. L., Klco, J. M., & Mullighan, C. G. (2020). Mechanistic insights and potential therapeutic approaches for NUP98-rearranged hematologic malignancies. *Blood, 136*(20), 2275-2289. doi:10.1182/blood.2020007093

Nacev, B. A., Jones, K. B., Intlekofer, A. M., Yu, J. S. E., Allis, C. D., Tap, W. D., . . . Nielsen, T. O. (2020). The epigenomics of sarcoma. *Nature Reviews Cancer, 20*(10), 608-623. doi:10.1038/s41568-020-0288-4

Nozawa, R. S., Yamamoto, T., Takahashi, M., Tachiwana, H., Maruyama, R., Hirota, T., & Saitoh, N. (2020). Nuclear microenvironment in cancer: Control through liquid-liquid phase separation. *Cancer Science, 111*(9), 3155-3163. doi:10.1111/cas.14551

Palumbo, E., Zhao, B., Xue, B., Uversky, V. N., & Dave, V. (2020). Analyzing aggregation propensities of clinically relevant PTEN mutants: a new culprit in pathogenesis of cancer and other PTENopathies. *Journal of Biomolecular Structure & Dynamics, 38*(8), 2253-2266. doi:10.1080/07391102.2019.1630005

Park, Y. J., Choi, D. W., Cho, S. W., Han, J., Yang, S., & Choi, C. Y. (2020). Stress Granule Formation Attenuates RACK1-Mediated Apoptotic Cell Death Induced by Morusin. *International Journal of Molecular Sciences, 21*(15), 13. doi:10.3390/ijms21155360

Pegoraro, S., Ros, G., Sgubin, M., Petrosino, S., Zambelli, A., Sgarra, R., & Manfioletti, G. (2020). Targeting the intrinsically disordered architectural High Mobility Group A (HMGA) oncoproteins in breast cancer: learning from the past to design future strategies. *Expert Opinion on Therapeutic Targets, 24*(10), 953-969. doi:10.1080/14728222.2020.1814738

Riggs, C. L., Kedersha, N., Ivanov, P., & Anderson, P. (2020). Mammalian stress granules and P bodies at a glance. *Journal of Cell Science, 133*(16), 9. doi:10.1242/jcs.242487

Sinha, S., & Thirumalai, D. (2020). Self-generated persistent random forces drive phase separation in growing tumors. *Journal of Chemical Physics, 153*(20), 6. doi:10.1063/5.0026590

Sukhanova, M. V., Singatulina, A. S., Pastre, D., & Lavrik, O. I. (2020). Fused in Sarcoma (FUS) in DNA Repair: Tango with Poly(ADP-ribose) Polymerase 1 and Compartmentalisation of Damaged DNA. *International Journal of Molecular Sciences, 21*(19), 18. doi:10.3390/ijms21197020

Sun, S., & Zhou, J. (2020). Phase separation as a therapeutic target in tight junction-associated human diseases. *Acta Pharmacologica Sinica, 41*(10), 1310-1313. doi:10.1038/s41401-020-0470-y

Tsang, B., Pritisanac, I., Scherer, S. W., Moses, A. M., & Forman-Kay, J. D. (2020). Phase Separation as a Missing Mechanism for Interpretation of Disease Mutations. *Cell, 183*(7), 1742-1756. doi:10.1016/j.cell.2020.11.050

Wang, F., Li, J., Fan, S. J., Jin, Z. G., & Huang, C. (2020). Targeting stress granules: A novel therapeutic strategy for human diseases. *Pharmacological Research, 161*, 16. doi:10.1016/j.phrs.2020.105143

Wang, W., Chen, Y. Q., Xu, A. X., Cai, M. Y., Cao, J., Zhu, H., . . . He, Q. J. (2020). Protein phase separation: A novel therapy for cancer? *British Journal of Pharmacology, 177*(22), 5008-5030. doi:10.1111/bph.15242

Wheeler, R. J. (2020). Therapeutics-how to treat phase separation-associated diseases. *Emerging Topics in Life Sciences, 4*(3), 331-342. doi:10.1042/etls20190176

Wolfe, K., Kamata, R., Coutinho, K., Inoue, T., & Sasaki, A. T. (2020). Metabolic Compartmentalization at the Leading Edge of Metastatic Cancer Cells. *Frontiers in Oncology, 10*, 9. doi:10.3389/fonc.2020.554272

Yao, Y., Tan, H. W., Liang, Z. L., Wu, G. Q., Xu, Y. M., & Lau, A. T. Y. (2020). The Impact of Coilin Nonsynonymous SNP Variants E121K and V145I on Cell Growth and Cajal Body Formation: The First Characterization. *Genes, 11*(8), 18. doi:10.3390/genes11080895

Yi, M., Tan, Y. X., Wang, L., Cai, J., Li, X. L., Zeng, Z. Y., . . . Xiang, B. (2020). TP63 links chromatin remodeling and enhancer reprogramming to epidermal differentiation and squamous cell carcinoma development. *Cellular and Molecular Life Sciences, 77*(21), 4325-4346. doi:10.1007/s00018-020-03539-2

Zelenka, T., & Spilianakis, C. (2020). SATB1-mediated chromatin landscape in T cells. *Nucleus, 11*(1), 117-131. doi:10.1080/19491034.2020.1775037

Zhang, H., Ji, X., Li, P. L., Liu, C., Lou, J. Z., Wang, Z., . . . Zhu, X. L. (2020). Liquid-liquid phase separation in biology: mechanisms, physiological functions and human diseases. *Science China-Life Sciences, 63*(7), 953-985. doi:10.1007/s11427-020-1702-x

Zhang, H. Y., Zhao, R. W., Tones, J., Liu, M. C., Dilley, R. L., Chenoweth, D. M., . . . Lampson, M. A. (2020). Nuclear body phase separation drives telomere clustering in ALT cancer cells. *Molecular Biology of the Cell, 31*(18), 2048-2056. doi:10.1091/mbc.E19-10-0589

Zhang, J. M., & Zou, L. (2020). Alternative lengthening of telomeres: from molecular mechanisms to therapeutic outlooks. *Cell and Bioscience, 10*(1), 9. doi:10.1186/s13578-020-00391-6

Zhang, J. Z., Lu, T. W., Stolerman, L. M., Tenner, B., Yang, J. R., Zhang, J. F., . . . Zhang, J. (2020). Phase Separation of a PKA Regulatory Subunit Controls cAMP Compartmentation and Oncogenic Signaling. *Cell, 182*(6), 1531-+. doi:10.1016/j.cell.2020.07.043

Ahmed, J., Meszaros, A., Lazar, T., & Tompa, P. (2021). DNA-binding domain as the minimal region driving RNA-dependent liquid-liquid phase separation of androgen receptor. *Protein Science, 30*(7), 1380-1392. doi:10.1002/pro.4100

Ahn, J. H., Davis, E. S., Daugird, T. A., Zhao, S., Quiroga, I. Y., Uryu, H., . . . Wang, G. G. (2021). Phase separation drives aberrant chromatin looping and cancer development. *Nature, 595*(7868), 591-+. doi:10.1038/s41586-021-03662-5

Bennett, Z. T., Li, S. X., Sumer, B. D., & Gao, J. M. (2021). Polyvalent design in the cGAS-STING pathway. *Seminars in Immunology, 56*, 11. doi:10.1016/j.smim.2021.101580

Biesaga, M., Frigole-Vivas, M., & Salvatella, X. (2021). Intrinsically disordered proteins and biomolecular condensates as drug targets. *Current Opinion in Chemical Biology, 62*, 90-100. doi:10.1016/j.cbpa.2021.02.009

Boija, A., Klein, I. A., & Young, R. A. (2021). Biomolecular Condensates and Cancer. *Cancer Cell, 39*(2), 174-192. doi:10.1016/j.ccell.2020.12.003

Boltsis, I., Grosveld, F., Giraud, G., & Kolovos, P. (2021). Chromatin Conformation in Development and Disease. *Frontiers in Cell and Developmental Biology, 9*, 27. doi:10.3389/fcell.2021.723859

Boon, R. (2021). Metabolic Fuel for Epigenetic: Nuclear Production Meets Local Consumption. *Frontiers in Genetics, 12*, 15. doi:10.3389/fgene.2021.768996

Bywaters, B. C., & Rivera, G. M. (2021). Nck adaptors at a glance. *Journal of Cell Science, 134*(18), 10. doi:10.1242/jcs.258965

Cai, D. F., Liu, Z., & Lippincott-Schwartz, J. (2021). Biomolecular Condensates and Their Links to Cancer Progression. *Trends in Biochemical Sciences, 46*(7), 535-549. doi:10.1016/j.tibs.2021.01.002

Campos-Melo, D., Hawley, Z. C. E., Droppelmann, C. A., & Strong, M. J. (2021). The Integral Role of RNA in Stress Granule Formation and Function. *Frontiers in Cell and Developmental Biology, 9*, 19. doi:10.3389/fcell.2021.621779

Chen, M., Foster, J. P., Lock, I. C., Leisenring, N. H., Daniel, A. R., Floyd, W., . . . Kirsch, D. G. (2021). Radiation-Induced Phosphorylation of a Prion-Like Domain Regulates Transformation by FUS-CHOP. *Cancer Research, 81*(19), 4939-4948. doi:10.1158/0008-5472.Can-20-1497

Cheng, Y. M., Xie, W., Pickering, B. F., Chu, K. L., Savino, A. M., Yang, X. J., . . . Kharas, M. G. (2021). N-6-Methyladenosine on mRNA facilitates a phase-separated nuclear body that suppresses myeloid leukemic differentiation. *Cancer Cell, 39*(7), 958-+. doi:10.1016/j.ccell.2021.04.017

Davis, R. B., Kaur, T., Moosa, M. M., & Banerjee, P. R. (2021). FUS oncofusion protein condensates recruit mSWI/SNF chromatin remodeler via heterotypic interactions between prion-like domains. *Protein Science, 30*(7), 1454-1466. doi:10.1002/pro.4127

Dong, L., Han, D., Meng, X. Y., Xu, M. C., Zheng, C. W., & Xia, Q. (2021). Activating Mutation of SHP2 Establishes a Tumorigenic Phonotype Through Cell-Autonomous and Non-Cell-Autonomous Mechanisms. *Frontiers in Cell and Developmental Biology, 9*, 14. doi:10.3389/fcell.2021.630712

Esposito, M., Fang, C., Cook, K. C., Park, N., Wei, Y., Spadazzi, C., . . . Kang, Y. B. (2021). TGF-beta-induced DACT1 biomolecular condensates repress Wnt signalling to promote bone metastasis. *Nature Cell Biology, 23*(3), 257-+. doi:10.1038/s41556-021-00641-w

Fan, X. J., Wang, Y. L., Zhao, W. W., Bai, S. M., Ma, Y., Yin, X. K., . . . Wan, X. B. (2021). NONO phase separation enhances DNA damage repair by accelerating nuclear EGFR-induced DNA-PK activation. *American Journal of Cancer Research, 11*(6), 2838-+.

Fang, Z. S., Zhang, Z., Liang, Z. J., Long, Z. R., Xiao, Y., Liang, Z. Y., . . . Huang, H. (2021). Liquid-Liquid Phase Separation-Related Genes Associated with Tumor Grade and Prognosis in Hepatocellular Carcinoma: A Bioinformatic Study. *International Journal of General Medicine, 14*, 9671-9679. doi:10.2147/ijgm.S342602

Fijen, C., & Rothenberg, E. (2021). The evolving complexity of DNA damage foci: RNA, condensates and chromatin in DNA double-strand break repair. *DNA Repair, 105*, 11. doi:10.1016/j.dnarep.2021.103170

Gebauer, F., Schwarzl, T., Valcarcel, J., & Hentze, M. W. (2021). RNA-binding proteins in human genetic disease. *Nature Reviews Genetics, 22*(3), 185-198. doi:10.1038/s41576-020-00302-y

Ghodke, I., Remisova, M., Furst, A., Kilic, S., Reina-San-Martin, B., Poetsch, A. R., . . . Soutoglou, E. (2021). AHNAK controls 53BP1-mediated p53 response by restraining 53BP1 oligomerization and phase separation. *Molecular Cell, 81*(12), 2596-+. doi:10.1016/j.molcel.2021.04.010

Guo, R. C., Zhang, X. H., Fan, P. S., Song, B. L., Li, Z. X., Duan, Z. Y., . . . Wang, H. (2021). In Vivo Self-Assembly Induced Cell Membrane Phase Separation for Improved Peptide Drug Internalization. *Angewandte Chemie-International Edition, 60*(47), 25128-25134. doi:10.1002/anie.202111839

Hosoya, Y., & Ohkanda, J. (2021). Intrinsically Disordered Proteins as Regulators of Transient Biological Processes and as Untapped Drug Targets. *Molecules, 26*(8), 14. doi:10.3390/molecules26082118

Ismail, H., Liu, X., Yang, F. R., Li, J. Y., Zahid, A., Dou, Z., . . . Yao, X. B. (2021). Mechanisms and regulation underlying membraneless organelle plasticity control. *Journal of Molecular Cell Biology, 13*(4), 239-258. doi:10.1093/jmcb/mjab028

Kanakamani, S., Suresh, P. S., & Venkatesh, T. (2021). Regulation of processing bodies: From viruses to cancer epigenetic machinery. *Cell Biology International, 45*(4), 708-719. doi:10.1002/cbin.11527

Kang, W. Y., Ferruzzi, J., Spatarelu, C. P., Han, Y. L., Sharma, Y., Koehler, S. A., . . . Fredberg, J. J. (2021). A novel jamming phase diagram links tumor invasion to non-equilibrium phase separation. *Iscience, 24*(11), 31. doi:10.1016/j.isci.2021.103252

Kim, G. H., & Kwon, I. (2021). Distinct roles of hnRNPH1 low-complexity domains in splicing and transcription. *Proceedings of the National Academy of Sciences of the United States of America, 118*(50), 10. doi:10.1073/pnas.2109668118

Kolonko-Adamska, M., Uversky, V. N., & Greb-Markiewicz, B. (2021). The Participation of the Intrinsically Disordered Regions of the bHLH-PAS Transcription Factors in Disease Development. *International Journal of Molecular Sciences, 22*(6), 33. doi:10.3390/ijms22062868

Lafontaine, D. L. J., Riback, J. A., Bascetin, R., & Brangwynne, C. P. (2021). The nucleolus as a multiphase liquid condensate. *Nature Reviews Molecular Cell Biology, 22*(3), 165-182. doi:10.1038/s41580-020-0272-6

Lezaja, A., & Altmeyer, M. (2021). Dealing with DNA lesions: When one cell cycle is not enough. *Current Opinion in Cell Biology, 70*, 27-36. doi:10.1016/j.ceb.2020.11.001

Li, R. H., Tian, T., Ge, Q. W., He, X. Y., Shi, C. Y., Li, J. H., . . . Lin, A. F. (2021). A phosphatidic acid-binding lncRNA SNHG9 facilitates LATS1 liquid-liquid phase separation to promote oncogenic YAP signaling. *Cell Research, 31*(10), 1088-1105. doi:10.1038/s41422-021-00530-9

Li, W. H., Jiang, C. W., & Zhang, E. H. (2021). Advances in the phase separation-organized membraneless organelles in cells: a narrative review. *Translational Cancer Research, 10*(11), 4929-4946. doi:10.21037/tcr-21-1111

Li, W. H., Wu, L., Jia, H., Lin, Z. H., Zhong, R. H., Li, Y. K., . . . Zhang, E. H. (2021). The low-complexity domains of the KMT2D protein regulate histone monomethylation transcription to facilitate pancreatic cancer progression. *Cellular & Molecular Biology Letters, 26*(1), 20. doi:10.1186/s11658-021-00292-7

Li, Y. W., Tang, W. H., & Guo, M. (2021). The cell as matter: Connecting molecular biology to cellular functions. *Matter, 4*(6), 1863-1891. doi:10.1016/j.matt.2021.03.013

Liu, J., Xie, Y., Guo, J., Li, X., Wang, J. J., Jiang, H. M., . . . Liu, Z. Q. (2021). Targeting NSD2-mediated SRC-3 liquid-liquid phase separation sensitizes bortezomib treatment in multiple myeloma. *Nature Communications, 12*(1), 14. doi:10.1038/s41467-021-21386-y

Liu, Q. X., Li, J. X., Zhang, W. J., Xiao, C., Zhang, S. H., Nian, C., . . . Zhou, D. W. (2021). Glycogen accumulation and phase separation drives liver tumor initiation. *Cell, 184*(22), 5559-+. doi:10.1016/j.cell.2021.10.001

Liu, S. Y., Wang, T. S., Shi, Y. L., Bai, L., Wang, S. S., Guo, D., . . . Liu, H. (2021). USP42 drives nuclear speckle mRNA splicing via directing dynamic phase separation to promote tumorigenesis. *Cell Death and Differentiation, 28*(8), 2482-2498. doi:10.1038/s41418-021-00763-6

Lu, B., Zou, C. Y., Yang, M. L., He, Y. Y., He, J. C., Zhang, C. X., . . . Zhao, W. (2021). Pharmacological Inhibition of Core Regulatory Circuitry Liquid-liquid Phase Separation Suppresses Metastasis and Chemoresistance in Osteosarcoma. *Advanced Science, 8*(20), 14. doi:10.1002/advs.202101895

Lu, J. H., Qian, J. J., Xu, Z. T., Yin, S. Y., Zhou, L., Zheng, S. S., & Zhang, W. (2021). Emerging Roles of Liquid-Liquid Phase Separation in Cancer: From Protein Aggregation to Immune-Associated Signaling. *Frontiers in Cell and Developmental Biology, 9*, 15. doi:10.3389/fcell.2021.631486

Luo, Y. Y., Wu, J. J., & Li, Y. M. (2021). Regulation of liquid-liquid phase separation with focus on post-translational modifications. *Chemical Communications, 57*(98), 13275-13287. doi:10.1039/d1cc05266g

Ma, X. Y., Ying, Y. F., Xie, H. Y., Liu, X. Y., Wang, X., & Li, J. F. (2021). The Regulatory Role of RNA Metabolism Regulator TDP-43 in Human Cancer. *Frontiers in Oncology, 11*, 10. doi:10.3389/fonc.2021.755096

McCluggage, F., & Fox, A. H. (2021). Paraspeckle nuclear condensates: Global sensors of cell stress? *Bioessays, 43*(5), 18. doi:10.1002/bies.202000245

Meng, F. S., Yu, Z. Y., Zhang, D., Chen, S. S., Guan, H. X., Zhou, R. Y., . . . Xu, P. L. (2021). Induced phase separation of mutant NF2 imprisons the cGAS-STING machinery to abrogate antitumor immunity. *Molecular Cell, 81*(20), 4147-+. doi:10.1016/j.molcel.2021.07.040

Naveed, A., Cooper, J. A., Li, R. H., Hubbard, A., Chen, J. W., Liu, T., . . . Fox, A. H. (2021). NEAT1 polyA-modulating antisense oligonucleotides reveal opposing functions for both long non-coding RNA isoforms in neuroblastoma. *Cellular and Molecular Life Sciences, 78*(5), 2213-2230. doi:10.1007/s00018-020-03632-6

Orti, F., Navarro, A. M., Rabinovich, A., Wodak, S. J., & Marino-Buslje, C. (2021). Insight into membraneless organelles and their associated proteins: Drivers, Clients and Regulators. *Computational and Structural Biotechnology Journal, 19*, 3964-3977. doi:10.1016/j.csbj.2021.06.042

Peng, P. H., Hsu, K. W., & Wu, K. J. (2021). Liquid-liquid phase separation (LLPS) in cellular physiology and tumor biology. *American Journal of Cancer Research, 11*(8), 3766-3776.

Peng, Q., Wang, L. J., Wang, J., Liu, C., Zheng, X., Zhang, X. Y., . . . Ma, J. (2021). Epstein-Barr virus EBNA2 phase separation regulates cancer-associated alternative RNA splicing patterns. *Clinical and Translational Medicine, 11*(8), 7. doi:10.1002/ctm2.504

Petronilho, E. C., Pedrote, M. M., Marques, M. A., Passos, Y. M., Mota, M. F., Jakobus, B., . . . Silva, J. L. (2021). Phase separation of p53 precedes aggregation and is affected by oncogenic mutations and ligands. *Chemical Science, 12*(21), 7334-7349. doi:10.1039/d1sc01739j

Qin, D., Wei, R., Zhu, S. T., Min, L., & Zhang, S. T. (2021). MiR-490-3p Silences CDK1 and Inhibits the Proliferation of Colon Cancer Through an LLPS-Dependent miRISC System. *Frontiers in Molecular Biosciences, 8*, 8. doi:10.3389/fmolb.2021.561678

Qin, Z., Sun, H. H., Yue, M. T., Pan, X. W., Chen, L., Feng, X. H., . . . Ji, H. B. (2021). Phase separation of EML4-ALK in firing downstream signaling and promoting lung tumorigenesis. *Cell Discovery, 7*(1), 11. doi:10.1038/s41421-021-00270-5

Qiu, Y., Pan, M., & Chen, X. M. (2021). A Liquid-Liquid Phase Separation-Related Gene Signature as Prognostic Biomarker for Epithelial Ovarian Cancer. *Frontiers in Oncology, 11*, 8. doi:10.3389/fonc.2021.671892

Reggiani, F., Gobbi, G., Ciarrocchi, A., & Sancisi, V. (2021). YAP and TAZ Are Not Identical Twins. *Trends in Biochemical Sciences, 46*(2), 154-168. doi:10.1016/j.tibs.2020.08.012

Sampson, J., Richards, M. W., Choi, J., Fry, A. M., & Bayliss, R. (2021). Phase-separated foci of EML4-ALK facilitate signalling and depend upon an active kinase conformation. *Embo Reports, 22*(12), 18. doi:10.15252/embr.202153693

Sanchez, A., Lee, D. H. Y., Kim, D. I., & Miller, K. M. (2021). Making Connections: Integrative Signaling Mechanisms Coordinate DNA Break Repair in Chromatin. *Frontiers in Genetics, 12*, 23. doi:10.3389/fgene.2021.747734

Scourzic, L., Salataj, E., & Apostolou, E. (2021). Deciphering the Complexity of 3D Chromatin Organization Driving Lymphopoiesis and Lymphoid Malignancies. *Frontiers in Immunology, 12*, 23. doi:10.3389/fimmu.2021.669881

Shi, B., Li, W., Song, Y. S., Wang, Z. J., Ju, R., Ulman, A., . . . Jiang, H. (2021). UTX condensation underlies its tumour-suppressive activity. *Nature, 597*(7878), 726-+. doi:10.1038/s41586-021-03903-7

Simanov, G., Dang, I., Fokin, A. I., Oguievetskaia, K., Campanacci, V., Cherfils, J., & Gautreau, A. M. (2021). Arpin Regulates Migration Persistence by Interacting with Both Tankyrases and the Arp2/3 Complex. *International Journal of Molecular Sciences, 22*(8), 16. doi:10.3390/ijms22084115

Sprunger, M. L., & Jackrel, M. E. (2021). Prion-Like Proteins in Phase Separation and Their Link to Disease. *Biomolecules, 11*(7), 18. doi:10.3390/biom11071014

Sugiura, R. (2021). Stress granules safeguard against MAPK signaling hyperactivation by sequestering PKC/Pck2: new findings and perspectives. *Current Genetics, 67*(6), 857-863. doi:10.1007/s00294-021-01192-1

Sunkel, B. D., & Stanton, B. Z. (2021). Pioneer factors in development and cancer. *Iscience, 24*(10), 21. doi:10.1016/j.isci.2021.103132

Takayama, K., Kosaka, T., Suzuki, T., Hongo, H., Oya, M., Fujimura, T., . . . Inoue, S. (2021). Subtype-specific collaborative transcription factor networks are promoted by OCT4 in the progression of prostate cancer. *Nature Communications, 12*(1), 16. doi:10.1038/s41467-021-23974-4

Tan, C. T., Chang, H. C., Zhou, Q. L., Yu, C. D., Fu, N. Y., Sabapathy, K., & Yu, V. C. (2021). MOAP-1-mediated dissociation of p62/SQSTM1 bodies releases Keap1 and suppresses Nrf2 signaling. *Embo Reports, 22*(1), 23.

Tateishi-Karimata, H., & Sugimoto, N. (2021). Roles of non-canonical structures of nucleic acids in cancer and neurodegenerative diseases. *Nucleic Acids Research, 49*(14), 7839-7855. doi:10.1093/nar/gkab580

Tchurikov, N. A., & Kravatsky, Y. V. (2021). The Role of rDNA Clusters in Global Epigenetic Gene Regulation. *Frontiers in Genetics, 12*, 11. doi:10.3389/fgene.2021.730633

Terlecki-Zaniewicz, S., Humer, T., Eder, T., Schmoellerl, J., Heyes, E., Manhart, G., . . . Grebien, F. (2021). Biomolecular condensation of NUP98 fusion proteins drives leukemogenic gene expression. *Nature Structural & Molecular Biology, 28*(2), 190-+. doi:10.1038/s41594-020-00550-w

Tong, X. Y., Quan, Y., & Zhang, H. Y. (2021). NUDT5 as a novel drug target and prognostic biomarker for ER-positive breast cancer. *Drug Discovery Today, 26*(3), 620-625. doi:10.1016/j.drudis.2020.11.031

Tulpule, A., Guan, J., Neel, D. S., Allegakoen, H. R., Lin, Y. P., Brown, D., . . . Bivona, T. G. (2021). Kinase-mediated RAS signaling via membraneless cytoplasmic protein granules. *Cell, 184*(10), 2649-+. doi:10.1016/j.cell.2021.03.031

Usher, E. T., Sabri, N., Rohac, R., Boal, A. K., Mittag, T., & Showalter, S. A. (2021). Intrinsically disordered substrates dictate SPOP subnuclear localization and ubiquitination activity. *Journal of Biological Chemistry, 296*, 16. doi:10.1016/j.jbc.2021.100693

Velazquez-Cruz, A., Banos-Jaime, B., Diaz-Quintana, A., De la Rosa, M. A., & Diaz-Moreno, I. (2021). Post-translational Control of RNA-Binding Proteins and Disease-Related Dysregulation. *Frontiers in Molecular Biosciences, 8*, 15. doi:10.3389/fmolb.2021.658852

Vitale, E., Gugnoni, M., & Ciarrocchi, A. (2021). No Need to Stick Together to Be Connected: Multiple Types of Enhancers' Networking. *Cancers, 13*(20), 16. doi:10.3390/cancers13205201

Wang, R. J., Cao, L. X., Thorne, R. F., Zhang, X. D., Li, J. M., Shao, F. M., . . . Wu, M. (2021). LncRNA GIRGL drives CAPRIN1-mediated phase separation to suppress glutaminase-1 translation under glutamine deprivation. *Science Advances, 7*(13), 18. doi:10.1126/sciadv.abe5708

Wei, Y. J., Luo, H. C., Yee, P. P., Zhang, L. J., Liu, Z. J., Zheng, H. Y., . . . Li, W. (2021). Paraspeckle Protein NONO Promotes TAZ Phase Separation in the Nucleus to Drive the Oncogenic Transcriptional Program. *Advanced Science, 8*(24), 17. doi:10.1002/advs.202102653

Wu, Q., Schapira, M., Arrowsmith, C. H., & Barsyte-Lovejoy, D. (2021). Protein arginine methylation: from enigmatic functions to therapeutic targeting. *Nature Reviews Drug Discovery, 20*(7), 509-530. doi:10.1038/s41573-021-00159-8

Yu, M., Peng, Z. X., Qin, M., Liu, Y., Wang, J. N., Zhang, C., . . . Sun, S. G. (2021). Interferon-gamma induces tumor resistance to anti-PD-1 immunotherapy by promoting YAP phase separation. *Molecular Cell, 81*(6), 1216-+. doi:10.1016/j.molcel.2021.01.010

Zeigler, T. M., Chung, M. C., Narayan, O. P., & Guan, J. (2021). Protein phase separation: physical models and phase-separation- mediated cancer signaling. *Advances in Physics-X, 6*(1), 21. doi:10.1080/23746149.2021.1936638

Zhang, J., Zeng, Y. Y., Xing, Y. P., Li, X. R., Zhou, L. Q., Hu, L., . . . Wu, M. (2021). Myristoylation-mediated phase separation of EZH2 compartmentalizes STAT3 to promote lung cancer growth. *Cancer Letters, 516*, 84-98. doi:10.1016/j.canlet.2021.05.035

Zhang, W. C., Liu, W., Jia, L. F., Chen, D. M., Chang, I., Lake, M., . . . Wang, C. Y. (2021). Targeting KDM4A epigenetically activates tumor-cell-intrinsic immunity by inducing DNA replication stress. *Molecular Cell, 81*(10), 2148-+. doi:10.1016/j.molcel.2021.02.038

Zhao, S., Allis, C. D., & Wang, G. G. (2021). The language of chromatin modification in human cancers. *Nature Reviews Cancer, 21*(7), 413-430. doi:10.1038/s41568-021-00357-x

Zhao, Y. G., Codogno, P., & Zhang, H. (2021). Machinery, regulation and pathophysiological implications of autophagosome maturation. *Nature Reviews Molecular Cell Biology, 22*(11), 733-750. doi:10.1038/s41580-021-00392-4

Zheng, W. L., Xia, N. W., Zhang, J. J., Chen, N. H., Meurens, F., Liu, Z. P., & Zhu, J. Z. (2021). How the Innate Immune DNA Sensing cGAS-STING Pathway Is Involved in Autophagy. *International Journal of Molecular Sciences, 22*(24), 14. doi:10.3390/ijms222413232

Zhou, W., Mohr, L., Maciejowski, J., & Kranzusch, P. J. (2021). cGAS phase separation inhibits TREX1-mediated DNA degradation and enhances cytosolic DNA sensing. *Molecular Cell, 81*(4), 739-+. doi:10.1016/j.molcel.2021.01.024

Zuo, L. Y., Zhang, G. W., Massett, M., Cheng, J., Guo, Z. C., Wang, L., . . . Qi, Z. (2021). Loci-specific phase separation of FET fusion oncoproteins promotes gene transcription. *Nature Communications, 12*(1), 16. doi:10.1038/s41467-021-21690-7

Adnane, S., Marino, A., & Leucci, E. (2022). LncRNAs in human cancers: signal from noise. *Trends in Cell Biology, 32*(7), 565-573. doi:10.1016/j.tcb.2022.01.006

Andres-Sanchez, N., Fisher, D., & Krasinska, L. (2022). Physiological functions and roles in cancer of the proliferation marker Ki-67. *Journal of Cell Science, 135*(11), 13. doi:10.1242/jcs.258932

Angosto-Bazarra, D., Molina-Lopez, C., & Pelegrin, P. (2022). Physiological and pathophysiological functions of NLRP6: pro- and anti-inflammatory roles. *Communications Biology, 5*(1), 8. doi:10.1038/s42003-022-03491-w

Backwell, L., & Marsh, J. A. (2022). Diverse Molecular Mechanisms Underlying Pathogenic Protein Mutations: Beyond the Loss-of-Function Paradigm. *Annual Review of Genomics and Human Genetics, 23*, 475-498. doi:10.1146/annurev-genom-111221-103208

Bakhshandeh, S., Werner, C., Fratzl, P., & Cipitria, A. (2022). Microenvironment-mediated cancer dormancy: Insights from metastability theory. *Proceedings of the National Academy of Sciences of the United States of America, 119*(1), 9. doi:10.1073/pnas.2111046118

Banani, S. F., Afeyan, L. K., Hawken, S. W., Henninger, J. E., Dall'Agnese, A., Clark, V. E., . . . Young, R. A. (2022). Genetic variation associated with condensate dysregulation in disease. *Developmental Cell, 57*(14), 1776-+. doi:10.1016/j.devcel.2022.06.010

Biancon, G., Joshi, P., Zimmer, J. T., Hunck, T., Gao, Y. M., Lessard, M. D., . . . Halene, S. (2022). Precision analysis of mutant U2AF1 activity reveals deployment of stress granules in myeloid malignancies. *Molecular Cell, 82*(6), 1107-+. doi:10.1016/j.molcel.2022.02.025

Bratek-Skicki, A., Van Nerom, M., Maes, D., & Tompa, P. (2022). Biological colloids: Unique properties of membraneless organelles in the cell. *Advances in Colloid and Interface Science, 310*, 15. doi:10.1016/j.cis.2022.102777

Chakravarty, A. K., McGrail, D. J., Lozanoski, T. M., Dunn, B. S., Shih, D. J. H., Cirillo, K. M., . . . Sahni, N. (2022). Biomolecular Condensation: A New Phase in Cancer Research. *Cancer Discovery, 12*(9), 2031-2043. doi:10.1158/2159-8290.Cd-21-1605

Chandra, B., Michmerhuizen, N. L., Shirnekhi, H. K., Tripathi, S., Pioso, B. J., Baggett, D. W., . . . Kriwacki, R. W. (2022). Phase Separation Mediates NUP98 Fusion Oncoprotein Leukemic Transformation. *Cancer Discovery, 12*(4), 1152-1169. doi:10.1158/2159-8290.Cd-21-0674

Chen, C., Fu, G. H., Guo, Q. Q., Xue, S., & Luo, S. Z. (2022). Phase separation of p53 induced by its unstructured basic region and prevented by oncogenic mutations in tetramerization domain. *International Journal of Biological Macromolecules, 222*, 207-216. doi:10.1016/j.ijbiomac.2022.09.087

Chen, S., Cao, X. F., Zhang, J. Y., Wu, W. Y., Zhang, B., & Zhao, F. Q. (2022). circVAMP3 Drives CAPRIN1 Phase Separation and Inhibits Hepatocellular Carcinoma by Suppressing c-Myc Translation. *Advanced Science, 9*(8), 14. doi:10.1002/advs.202103817

Chu, X. Y., Xu, Y. Y., Tong, X. Y., Wang, G., & Zhang, H. Y. (2022). The Legend of ATP: From Origin of Life to Precision Medicine. *Metabolites, 12*(5), 9. doi:10.3390/metabo12050461

Dai, Z. J., Li, G. L., Chen, Q. Y., & Yang, X. R. (2022). Ser392 phosphorylation modulated a switch between p53 and transcriptional condensates. *Biochimica Et Biophysica Acta-Gene Regulatory Mechanisms, 1865*(4), 10. doi:10.1016/j.bbagrm.2022.194827

Damizia, M., Altieri, L., & Lavia, P. (2022). Non-transport roles of nuclear import receptors: In need of the right balance. *Frontiers in Cell and Developmental Biology, 10*, 9. doi:10.3389/fcell.2022.1041938

Davis, R. B., Moosa, M. M., & Banerjee, P. R. (2022). Ectopic biomolecular phase transitions: fusion proteins in cancer pathologies. *Trends in Cell Biology, 32*(8), 681-695. doi:10.1016/j.tcb.2022.03.005

Debek, S., & Juszczynski, P. (2022). Super enhancers as master gene regulators in the pathogenesis of hematologic malignancies. *Biochimica Et Biophysica Acta-Reviews on Cancer, 1877*(2), 9. doi:10.1016/j.bbcan.2022.188697

Dey, A., Sen, S., & Maulik, U. (2022). Study of transcription factor druggabilty for prostate cancer using structure information, gene regulatory networks and protein moonlighting. *Briefings in Bioinformatics, 23*(1), 13. doi:10.1093/bib/bbab465

Enenkel, C., Kang, R. W., Wilfling, F., & Ernst, O. P. (2022). Intracellular localization of the proteasome in response to stress conditions. *Journal of Biological Chemistry, 298*(7), 10. doi:10.1016/j.jbc.2022.102083

Ferretti, G. D. S., Quarti, J., dos Santos, G., Rangel, L. P., & Silva, J. L. (2022). Anticancer Therapeutic Strategies Targeting p53 Aggregation. *International Journal of Molecular Sciences, 23*(19), 19. doi:10.3390/ijms231911023

Fonteneau, G., Redding, A., Hoag-Lee, H., Sim, E. S., Heinrich, S., Gaida, M. M., & Grabocka, E. (2022). Stress Granules Determine the Development of Obesity-Associated Pancreatic Cancer. *Cancer Discovery, 12*(8), 1984-2005. doi:10.1158/2159-8290.Cd-21-1672

Gao, Y. F., Jiang, M., Guo, F. Q., Liu, X. J., Zhang, Q., Yang, S., . . . Dong, Z. G. (2022). A novel lncRNA MTAR1 promotes cancer development through IGF2BPs mediated post-transcriptional regulation of c-MYC. *Oncogene, 41*(42), 4736-4753. doi:10.1038/s41388-022-02464-x

Gonzalez-Arzola, K., Diaz-Quintana, A., Bernardo-Garcia, N., Martinez-Fabregas, J., Rivero-Rodriguez, F., Casado-Combreras, M. A., . . . Diaz-Moreno, I. (2022). Nucleus-translocated mitochondrial cytochrome c liberates nucleophosmin-sequestered ARF tumor suppressor by changing nucleolar liquid-liquid phase separation. *Nature Structural & Molecular Biology, 29*(10), 1024-+. doi:10.1038/s41594-022-00842-3

Gu, X., Zhuang, A., Yu, J., Chai, P. W., Jia, R. B., & Ruan, J. (2022). Phase separation drives tumor pathogenesis and evolution: all roads lead to Rome. *Oncogene, 41*(11), 1527-1535. doi:10.1038/s41388-022-02195-z

Han, Z. Z., & Li, W. (2022). Enhancer RNA: What we know and what we can achieve. *Cell Proliferation, 55*(4), 10. doi:10.1111/cpr.13202

Hanselmann, R. G., & Welter, C. (2022). Origin of Cancer: Cell work is the Key to Understanding Cancer Initiation and Progression. *Frontiers in Cell and Developmental Biology, 10*, 14. doi:10.3389/fcell.2022.787995

Huang, D., & Kraus, W. L. (2022). The expanding universe of PARP1-mediated molecular and therapeutic mechanisms. *Molecular Cell, 82*(12), 2315-2334. doi:10.1016/j.molcel.2022.02.021

Huang, N., Dong, H., & Shao, B. (2022). Phase separation in immune regulation and immune-related diseases. *J Mol Med (Berl), 100*(10), 1427-1440. doi:10.1007/s00109-022-02253-9

Igelmann, S., Lessard, F., & Ferbeyre, G. (2022). Liquid-Liquid Phase Separation in Cancer Signaling, Metabolism and Anticancer Therapy. *Cancers, 14*(7), 12. doi:10.3390/cancers14071830

Ito, S., Das, N. D., Umehara, T., & Koseki, H. (2022). Factors and Mechanisms That Influence Chromatin-Mediated Enhancer-Promoter Interactions and Transcriptional Regulation. *Cancers, 14*(21), 16. doi:10.3390/cancers14215404

Jia, Z. X., Yang, S. X., Li, M. Y., Lei, Z. Y., Ding, X., Fan, M. J., . . . Yan, Q. F. (2022). A novel NF2 splicing mutant causes neurofibromatosis type 2 via liquid-liquid phase separation with large tumor suppressor and Hippo pathway. *Iscience, 25*(11), 25. doi:10.1016/j.isci.2022.105275

Jiang, A., Zhang, S. W., Wang, X. Y., & Li, D. (2022). RBM15 condensates modulate m(6)A modification of STYK1 to promote tumorigenesis. *Computational and Structural Biotechnology Journal, 20*, 4825-4836. doi:10.1016/j.csbj.2022.08.068

Jin, X. J., Zhou, M., Chen, S. X., Li, D. Q., Cao, X. L., & Liu, B. D. (2022). Effects of pH alterations on stress- and aging-induced protein phase separation. *Cellular and Molecular Life Sciences, 79*(7), 23. doi:10.1007/s00018-022-04393-0

Kabra, A., & Bushweller, J. (2022). The Intrinsically Disordered Proteins MLLT3 (AF9) and MLLT1 (ENL)-Multimodal Transcriptional Switches With Roles in Normal Hematopoiesis, MLL Fusion Leukemia, and Kidney Cancer. *Journal of Molecular Biology, 434*(1), 11. doi:10.1016/j.jmb.2021.167117

Lee, J., Cho, H., & Kwon, I. (2022). Phase separation of low-complexity domains in cellular function and disease. *Experimental and Molecular Medicine, 54*(9), 1412-1422. doi:10.1038/s12276-022-00857-2

Li, J., Ye, Y. Y., Liu, Z. H., Zhang, G. Y., Dai, H. Q., Li, J. Q., . . . Su, S. C. (2022). Macrophage mitochondrial fission improves cancer cell phagocytosis induced by therapeutic antibodies and is impaired by glutamine competition. *Nature Cancer, 3*(4), 453-+. doi:10.1038/s43018-022-00354-5

Li, J., Zhao, H. C., McMahon, A., & Yan, S. (2022). APE1 assembles biomolecular condensates to promote the ATR-Chk1 DNA damage response in nucleolus. *Nucleic Acids Research, 50*(18), 10503-10525. doi:10.1093/nar/gkac853

Li, M., Li, M. Y., Xia, Y., Li, G. S., Su, X. H., Wang, D. M., . . . Ji, C. Y. (2022). HDAC1/3-dependent moderate liquid-liquid phase separation of YY1 promotes METTL3 expression and AML cell proliferation. *Cell Death & Disease, 13*(11), 12. doi:10.1038/s41419-022-05435-y

Li, X. M., Wu, H. L., Xia, Q. D., Zhou, P., Wang, S. G., Yu, X., & Hu, J. (2022). Novel insights into the SPOP E3 ubiquitin ligase: From the regulation of molecular mechanisms to tumorigenesis. *Biomedicine & Pharmacotherapy, 149*, 13. doi:10.1016/j.biopha.2022.112882

Liebl, M. C., & Hofmann, T. G. (2022). Regulating the p53 Tumor Suppressor Network at PML Biomolecular Condensates. *Cancers, 14*(19), 18. doi:10.3390/cancers14194549

Loh, D., & Reiter, R. J. (2022). Melatonin: Regulation of Prion Protein Phase Separation in Cancer Multidrug Resistance. *Molecules, 27*(3), 62. doi:10.3390/molecules27030705

Lu, X. Y., Wang, J., Wang, W., Lu, C. F., Qu, T. Y., He, X. Z., . . . Zhang, E. B. (2022). Copy number amplification and SP1-activated lncRNA MELTF-AS1 regulates tumorigenesis by driving phase separation of YBX1 to activate ANXA8 in non-small cell lung cancer. *Oncogene, 41*(23), 3222-3238. doi:10.1038/s41388-022-02292-z

Luo, X. J., He, M. M., Liu, J., Zheng, J. B., Wu, Q. N., Chen, Y. X., . . . Luo, H. Y. (2022). LncRNA TMPO-AS1 promotes esophageal squamous cell carcinoma progression by forming biomolecular condensates with FUS and p300 to regulate TMPO transcription. *Experimental and Molecular Medicine, 54*(6), 834-847. doi:10.1038/s12276-022-00791-3

Luo, Y., Xiang, S. S., & Feng, J. B. (2022). Protein Phase Separation: New Insights into Carcinogenesis. *Cancers, 14*(23), 22. doi:10.3390/cancers14235971

Marques, M. A., de Oliveira, G. A. P., & Silva, J. L. (2022). The chameleonic behavior of p53 in health and disease: the transition from a client to an aberrant condensate scaffold in cancer. *Essays in Biochemistry, 66*(7), 1023-1033. doi:10.1042/ebc20220064

Mehta, S., & Zhang, J. (2022). Liquid-liquid phase separation drives cellular function and dysfunction in cancer. *Nature Reviews Cancer, 22*(4), 239-252. doi:10.1038/s41568-022-00444-7

Meszaros, A., Ahmed, J., Russo, G., Tompa, P., & Lazar, T. (2022). The evolution and polymorphism of mono-amino acid repeats in androgen receptor and their regulatory role in health and disease. *Frontiers in Medicine, 9*, 22. doi:10.3389/fmed.2022.1019803

Nsengimana, B., Khan, F. A., Awan, U. A., Wang, D. D., Fang, N., Wei, W. Q., . . . Ji, S. P. (2022). Pseudogenes and Liquid Phase Separation in Epigenetic Expression. *Frontiers in Oncology, 12*, 11. doi:10.3389/fonc.2022.912282

Nsengimana, B., Khan, F. A., Ngowi, E. E., Zhou, X. F., Jin, Y., Jia, Y. T., . . . Ji, S. P. (2022). Processing body (P-body) and its mediators in cancer. *Molecular and Cellular Biochemistry, 477*(4), 1217-1238. doi:10.1007/s11010-022-04359-7

Pei, H. X., Guo, W. K., Peng, Y. R., Xiong, H., & Chen, Y. H. (2022). Targeting key proteins involved in transcriptional regulation for cancer therapy: Current strategies and future prospective. *Medicinal Research Reviews, 42*(4), 1607-1660. doi:10.1002/med.21886

Peng, Q., Tan, S. M., Xia, L. Z., Wu, N. Y., Oyang, L., Tang, Y. Y., . . . Liao, Q. J. (2022). Phase separation in Cancer: From the Impacts and Mechanisms to Treatment potentials. *International Journal of Biological Sciences, 18*(13), 5103-5122. doi:10.7150/ijbs.75410

Puca, F., Fedele, M., Rasio, D., & Battista, S. (2022). Role of Diet in Stem and Cancer Stem Cells. *International Journal of Molecular Sciences, 23*(15), 28. doi:10.3390/ijms23158108

Quiroga, I. Y., Ahn, J. H., Wang, G. G., & Phanstiel, D. (2022). Oncogenic fusion proteins and their role in three-dimensional chromatin structure, phase separation, and cancer. *Current Opinion in Genetics & Development, 74*, 8. doi:10.1016/j.gde.2022.101901

Ren, J., Zhang, Z. Y., Zong, Z., Zhang, L., & Zhou, F. F. (2022). Emerging Implications of Phase Separation in Cancer. *Advanced Science, 9*(31), 23. doi:10.1002/advs.202202855

Roggero, C. M., Esser, V., Duan, L. L., Rice, A. M., Ma, S. H., Raj, G. V., . . . Rizoi, J. (2022). Poly-glutamine-dependent self-association as a potential mechanism for regulation of androgen receptor activity. *Plos One, 17*(1), 20. doi:10.1371/journal.pone.0258876

Rubio-Ramos, A., Bernabe-Rubio, M., Labat-de-Hoz, L., Casares-Arias, J., Kremer, L., Correas, I., & Alonso, M. A. (2022). MALL, a membrane-tetra-spanning proteolipid overexpressed in cancer, is present in membraneless nuclear biomolecular condensates. *Cellular and Molecular Life Sciences, 79*(5), 15. doi:10.1007/s00018-022-04270-w

Schmidt, H. B., Jaafar, Z. A., Wulff, B. E., Rodencal, J. J., Hong, K. B., Aziz-Zanjani, M. O., . . . Brandman, O. (2022). Oxaliplatin disrupts nucleolar function biophysical. *Cell Reports, 41*(6), 22. doi:10.1016/j.celrep.2022.111629

Schwed-Gross, A., Hamiel, H., Faber, G. P., Angel, M., Ben-Yishay, R., Benichou, J. I. C., . . . Shav-Tal, Y. (2022). Glucocorticoids enhance chemotherapy-driven stress granule assembly and impair granule dynamics, leading to cell death. *Journal of Cell Science, 135*(14), 19. doi:10.1242/jcs.259629

Sehgal, P. B. (2022). Interleukin-6 at the Host-Tumor Interface: STAT3 in Biomolecular Condensates in Cancer Cells. *Cells, 11*(7), 15. doi:10.3390/cells11071164

Shao, X. J., Chen, Y. Q., Xu, A. X., Xiang, D. Y., Wang, W., Du, W. X., . . . Ying, M. D. (2022). Deneddylation of PML/RAR alpha reconstructs functional PML nuclear bodies via orchestrating phase separation to eradicate APL. *Cell Death and Differentiation, 29*(8), 1654-1668. doi:10.1038/s41418-022-00955-8

Shi, J., Chen, S. Y., Shen, X. T., Yin, X. K., Zhao, W. W., Bai, S. M., . . . Fan, X. J. (2022). NOP53 undergoes liquid-liquid phase separation and promotes tumor radio-resistance. *Cell Death Discovery, 8*(1), 10. doi:10.1038/s41420-022-01226-8

Sideris, N., Dama, P., Bayraktar, S., Stiff, T., & Castellano, L. (2022). LncRNAs in breast cancer: a link to future approaches. *Cancer Gene Ther, 29*(12), 1866-1877. doi:10.1038/s41417-022-00487-w

Silva, J. L., Vieira, T. C., Cordeiro, Y., & de Oliveira, G. A. P. (2022). Nucleic acid actions on abnormal protein aggregation, phase transitions and phase separation. *Current Opinion in Structural Biology, 73*, 12. doi:10.1016/j.sbi.2022.102346

Somasekharan, S. P., Saxena, N., Zhang, F., Beraldi, E., Huang, J. N., Gentle, C., . . . Gleave, M. (2022). Regulation of AR mRNA translation in response to acute AR pathway inhibition. *Nucleic Acids Research, 50*(2), 1069-1091. doi:10.1093/nar/gkab1247

Somasundaram, K., Gupta, B., Jain, N., & Jana, S. (2022). LncRNAs divide and rule: The master regulators of phase separation. *Frontiers in Genetics, 13*, 12. doi:10.3389/fgene.2022.930792

Song, L. L., Yao, X. Y., Li, H. P., Peng, B., Boka, A. P., Liu, Y. M., . . . Wan, L. L. (2022). Hotspot mutations in the structured ENL YEATS domain link aberrant transcriptional condensates and cancer. *Molecular Cell, 82*(21), 4080-+. doi:10.1016/j.molcel.2022.09.034

Su, Y. Y., Chen, X. Q., Wang, H., Sun, L. L., Xu, Y., & Li, D. (2022). Enhancing cell membrane phase separation for inhibiting cancer metastasis with a stimuli-responsive DNA nanodevice. *Chemical Science, 13*(21), 6303-6308. doi:10.1039/d2sc00371f

Sun, L., Liu, X. P., Yan, X., Wu, S. J., Tang, X. Y., Chen, C., . . . Li, S. (2022). Identification of molecular subtypes based on liquid-liquid phase separation and cross-talk with immunological phenotype in bladder cancer. *Frontiers in Immunology, 13*, 20. doi:10.3389/fimmu.2022.1059568

Suzuki, H. I., & Onimaru, K. (2022). Biomolecular condensates in cancer biology. *Cancer Science, 113*(2), 382-391. doi:10.1111/cas.15232

Takayama, K. I., & Inoue, S. (2022). Targeting phase separation on enhancers induced by transcription factor complex formations as a new strategy for treating drug-resistant cancers. *Frontiers in Oncology, 12*, 15. doi:10.3389/fonc.2022.1024600

Taniue, K., & Akimitsu, N. (2022). Aberrant phase separation and cancer. *Febs Journal, 289*(1), 17-39. doi:10.1111/febs.15765

Tessier, S., Ferhi, O., Geoffroy, M. C., Gonzalez-Prieto, R., Canat, A., Quentin, S., . . . Lallemand-Breitenbach, V. (2022). Exploration of nuclear body-enhanced sumoylation reveals that PML represses 2-cell features of embryonic stem cells. *Nature Communications, 13*(1), 15. doi:10.1038/s41467-022-33147-6

Tong, X. H., Tang, R., Xu, J., Wang, W., Zhao, Y. J., Yu, X. J., & Shi, S. (2022). Liquid-liquid phase separation in tumor biology. *Signal Transduction and Targeted Therapy, 7*(1), 22. doi:10.1038/s41392-022-01076-x

Vu, T. Q., Peruzzi, J. A., Sant'Anna, L. E., Roth, E. W., & Kamat, N. P. (2022). Lipid Phase Separation in Vesicles Enhances TRAIL-Mediated Cytotoxicity. *Nano Letters, 22*(7), 2627-2634. doi:10.1021/acs.nanolett.1c04365

Wang, A. F., Abulaiti, X., Zhang, H., Su, H., Liu, G. Z., Gao, S. R., & Li, L. S. (2022). Cancer Cells Evade Stress-Induced Apoptosis by Promoting HSP70-Dependent Clearance of Stress Granules. *Cancers, 14*(19), 15. doi:10.3390/cancers14194671

Wang, D., Ye, R., Cai, Z. K., & Xue, Y. C. (2022). Emerging roles of RNA-RNA interactions in transcriptional regulation. *Wiley Interdisciplinary Reviews-Rna, 13*(5), 17. doi:10.1002/wrna.1712

Wang, H., Li, B. Y., Zuo, L. Y., Wang, B., Yan, Y., Tian, K., . . . Ji, X. (2022). The transcriptional coactivator RUVBL2 regulates Pol II clustering with diverse transcription factors. *Nature Communications, 13*(1), 26. doi:10.1038/s41467-022-33433-3

Wang, J. H., Gan, Y. X., Cao, J., Dong, X. F., & Ouyang, W. (2022). Pathophysiology of stress granules: An emerging link to diseases (Review). *International Journal of Molecular Medicine, 49*(4), 10. doi:10.3892/ijmm.2022.5099

Wang, J. Y., Meng, F., & Mao, F. (2022). Single cell sequencing analysis and transcriptome analysis constructed the liquid-liquid phase separation(LLPS)-related prognostic model for endometrial cancer. *Frontiers in Oncology, 12*, 12. doi:10.3389/fonc.2022.1005472

Wang, W. M., Qiao, S. Y., Li, G. Y., Cheng, J. H., Yang, C. C., Zhong, C., . . . Sui, G. C. (2022). A histidine cluster determines YY1-compartmentalized coactivators and chromatin elements in phase-separated enhancer clusters. *Nucleic Acids Research, 50*(9), 4917-4937. doi:10.1093/nar/gkac233

Wang, Z. Z., Zhai, Z. Y., Chen, C. Y., Tian, X. J., Xing, Z., Xing, P. F., . . . Dong, L. (2022). Air pollution particles hijack peroxidasin to disrupt immunosurveillance and promote lung cancer. *Elife, 11*, 31. doi:10.7554/eLife.75345

Wei, C. Z., Li, M. D., Li, X. M., Lyu, J. X., & Zhu, X. (2022). Phase Separation: "The Master Key" to Deciphering the Physiological and Pathological Functions of Cells. *Advanced Biology, 6*(7), 19. doi:10.1002/adbi.202200006

Wilkinson, E., Cui, Y. H., & He, Y. Y. (2022). Roles of RNA Modifications in Diverse Cellular Functions. *Frontiers in Cell and Developmental Biology, 10*, 26. doi:10.3389/fcell.2022.828683

Xiao, C. C., Wu, G. J., Chen, P. F., Gao, L. J., Chen, G. B., & Zhang, H. Y. (2022). Phase separation in epigenetics and cancer stem cells. *Frontiers in Oncology, 12*, 13. doi:10.3389/fonc.2022.922604

Xie, J. J., He, H., Kong, W. N., Li, Z. W., Gao, Z. T., Xie, D. Q., . . . Zhu, G. Y. (2022). Targeting androgen receptor phase separation to overcome antiandrogen resistance. *Nature Chemical Biology, 18*(12), 1341-+. doi:10.1038/s41589-022-01151-y

Xing, Z., Xue, J., Ma, X. D., Han, C. W., Wang, Z. Z., Luo, S. H., . . . Zhang, J. F. (2022). Intracellular mRNA phase separation induced by cationic polymers for tumor immunotherapy. *Journal of Nanobiotechnology, 20*(1), 16. doi:10.1186/s12951-022-01647-8

Yi, F., Cai, C., Ruan, B., Hao, M., Yeo, S. K., Haas, M., . . . Guan, J. L. (2022). Regulation of RB1CC1/FIP200 stability and autophagy function by CREBBP-mediated acetylation in an intrinsically disordered region. *Autophagy*, 1-16. doi:10.1080/15548627.2022.2148432

Yin, Q. Y., Zheng, M., Luo, Q. M., Jiang, D. W., Zhang, H. F., & Chen, C. S. (2022). YB-1 as an Oncoprotein: Functions, Regulation, Post-Translational Modifications, and Targeted Therapy. *Cells, 11*(7), 23. doi:10.3390/cells11071217

Yu-Qing, H., Peng-Ping, L., Ke, S., Ke-Xing, Y., Wei-Jun, Z., & Zhen-Yu, W. (2022). Comprehensive analysis of liquid-liquid phase separation-related genes in prediction of breast cancer prognosis. *Frontiers in Genetics, 13*, 15. doi:10.3389/fgene.2022.834471

Yuan, B. L., Zhou, X., Suzuki, K., Ramos-Mandujano, G., Wang, M. G., Tehseen, M., . . . Belmonte, J. C. I. (2022). Wiskott-Aldrich syndrome protein forms nuclear condensates and regulates alternative splicing. *Nature Communications, 13*(1), 20. doi:10.1038/s41467-022-31220-8

Zhang, L., Wang, S. B., Wang, W. M., Shi, J. M., Stovall, D. B., Li, D. D., & Sui, G. C. (2022). Phase-Separated Subcellular Compartmentation and Related Human Diseases. *International Journal of Molecular Sciences, 23*(10), 23. doi:10.3390/ijms23105491

Zhang, Y. X., Li, J., Feng, D., Peng, X. B., Wang, B., Han, T., & Zhang, Y. Y. (2022). Systematic Analysis of Molecular Characterization and Clinical Relevance of Liquid-Liquid Phase Separation Regulators in Digestive System Neoplasms. *Frontiers in Cell and Developmental Biology, 9*, 18. doi:10.3389/fcell.2021.820174

Zhao, J. Y., Xie, W., Yang, Z. C., Zhao, M., Ke, T., Xu, C. Q., . . . Wang, Q. K. (2022). Identification and characterization of a special type of subnuclear structure: AGGF1-coated paraspeckles. *Faseb Journal, 36*(6), 26. doi:10.1096/fj.202101690RR

Zheng, J. L., Wu, Z. P., Qiu, Y., Wang, X., & Jiang, X. B. (2022). An integrative multi-omics analysis based on liquid-liquid phase separation delineates distinct subtypes of lower-grade glioma and identifies a prognostic signature. *Journal of Translational Medicine, 20*(1), 22. doi:10.1186/s12967-022-03266-1

Zhou, T. Y., & Feng, Q. (2022). Androgen receptor signaling and spatial chromatin organization in castration-resistant prostate cancer. *Frontiers in Medicine, 9*, 10. doi:10.3389/fmed.2022.924087

Zhu, Q. Q., Zhang, C. G., Qu, T. Y., Lu, X. Y., He, X. Z., Li, W., . . . Zhang, E. R. (2022). MNX1-AS1 Promotes Phase Separation of IGF2BP1 to Drive c-Myc-Mediated Cell-Cycle Progression and Proliferation in Lung Cancer. *Cancer Research, 82*(23), 4340-4358. doi:10.1158/0008-5472.Can-22-1289

Zhuge, L. D., Zhang, K., Zhang, Z. L., Guo, W. T., Li, Y., & Bao, Q. (2022). A novel model based on liquid-liquid phase separation-Related genes correlates immune microenvironment profiles and predicts prognosis of lung squamous cell carcinoma. *Journal of Clinical Laboratory Analysis, 36*(1), 9. doi:10.1002/jcla.24135

Zou, H. Z., Pan, T., Gao, Y. Y., Chen, R. W., Li, S., Guo, J., . . . Li, Y. S. (2022). Pan-cancer assessment of mutational landscape in intrinsically disordered hotspots reveals potential driver genes. *Nucleic Acids Research, 50*(9), 13. doi:10.1093/nar/gkac028

Cuartero, S., Stik, G., & Stadhouders, R. (2023). Three-dimensional genome organization in immune cell fate and function. *Nature Reviews Immunology, 23*(4), 206-221. doi:10.1038/s41577-022-00774-5

Kato, H., Tateishi, K., Iwadate, D., Yamamoto, K., Fujiwara, H., Nakatsuka, T., . . . Fujishiro, M. (2023). HNF1B-driven three-dimensional chromatin structure for molecular classification in pancreatic cancers. *Cancer Science, 114*(4), 1672-1685. doi:10.1111/cas.15690

Sanya, D. R. A., Cava, C., & Onesime, D. (2023). Roles of RNA-binding proteins in neurological disorders, COVID-19, and cancer. *Human Cell, 36*(2), 493-514. doi:10.1007/s13577-022-00843-w

Zhang, F., Biswas, M., Massah, S., Lee, J., Lingadahalli, S., Wong, S., . . . Lallous, N. (2023). Dynamic phase separation of the androgen receptor and its coactivators key to regulate gene expression. *Nucleic Acids Research, 51*(1), 99-116. doi:10.1093/nar/gkac1158

Zhang, S., Cooper, J. A. L., Chong, Y. S., Naveed, A., Mayoh, C., Jayatilleke, N., . . . Fox, A. H. (2023). NONO enhances mRNA processing of super-enhancer-associated GATA2 and HAND2 genes in neuroblastoma. *Embo Reports, 24*(2), 19. doi:10.15252/embr.202254977
